# Dynamic genome-scale metabolic modeling of the yeast *Pichia pastoris*

**DOI:** 10.1186/s12918-017-0408-2

**Published:** 2017-02-21

**Authors:** Francisco Saitua, Paulina Torres, José Ricardo Pérez-Correa, Eduardo Agosin

**Affiliations:** Department of Chemical and Bioprocess Engineering, School of Engineering, Pontificia Universidad Católica de Chile, Avenida Vicuña Mackenna 4860, Santiago, Chile

**Keywords:** dFBA, *Pichia pastoris*, Pre/post regression diagnostics, Sensitivity, Identifiability, Significance, Genome-scale metabolic modeling, Fed-batch, MOMA, Bioprocess optimization, Reparametrization

## Abstract

**Background:**

*Pichia pastoris* shows physiological advantages in producing recombinant proteins, compared to other commonly used cell factories. This yeast is mostly grown in dynamic cultivation systems, where the cell’s environment is continuously changing and many variables influence process productivity. In this context, a model capable of explaining and predicting cell behavior for the rational design of bioprocesses is highly desirable. Currently, there are five genome-scale metabolic reconstructions of *P. pastoris* which have been used to predict extracellular cell behavior in stationary conditions.

**Results:**

In this work, we assembled a dynamic genome-scale metabolic model for glucose-limited, aerobic cultivations of *Pichia pastoris*. Starting from an initial model structure for batch and fed-batch cultures, we performed pre/post regression diagnostics to ensure that model parameters were identifiable, significant and sensitive. Once identified, the non-relevant ones were iteratively fixed until a priori robust modeling structures were found for each type of cultivation. Next, the robustness of these reduced structures was confirmed by calibrating the model with new datasets, where no sensitivity, identifiability or significance problems appeared in their parameters. Afterwards, the model was validated for the prediction of batch and fed-batch dynamics in the studied conditions.

Lastly, the model was employed as a case study to analyze the metabolic flux distribution of a fed-batch culture and to unravel genetic and process engineering strategies to improve the production of recombinant Human Serum Albumin (HSA). Simulation of single knock-outs indicated that deviation of carbon towards cysteine and tryptophan formation improves HSA production. The deletion of methylene tetrahydrofolate dehydrogenase could increase the HSA volumetric productivity by 630%. Moreover, given specific bioprocess limitations and strain characteristics, the model suggests that implementation of a decreasing specific growth rate during the feed phase of a fed-batch culture results in a 25% increase of the volumetric productivity of the protein.

**Conclusion:**

In this work, we formulated a dynamic genome scale metabolic model of *Pichia pastoris* that yields realistic metabolic flux distributions throughout dynamic cultivations. The model can be calibrated with experimental data to rationally propose genetic and process engineering strategies to improve the performance of a *P. pastoris* strain of interest.

**Electronic supplementary material:**

The online version of this article (doi:10.1186/s12918-017-0408-2) contains supplementary material, which is available to authorized users.

## Background

Recombinant protein production is a multibillion-dollar business, mainly comprised by therapeutic agents (i.e. recombinant biologic drugs) and industrial enzymes [1–3]. These compounds are commonly synthesized in *Escherichia coli*, *Saccharomyces cerevisiae* and Chinese Hamster Ovary cells (CHO) [1, 4–6]; however, there is strong pressure to find cost-effective alternatives to overcome technical and economic disadvantages of the aforementioned cell factories, especially in downstream processing [7].

Among the unconventional cell factories used for recombinant protein production, the methylotrophic yeast *Pichia pastoris* (syn. *Komagataella phaffii*) has received special attention thanks to its convenient physiology and easy handling [8]. There are strong promoters for this cell factory which are commercially available and that allow for the controlled expression of heterologous proteins [8]. Unlike *E. coli*, *P. pastoris* naturally performs post-translational modifications [6, 9], which are essential for most eukaryotic protein functionality [7, 10, 11]. In contrast to *S. cerevisiae*, *P. pastoris* exhibits a Crabtree-negative phenotype, showing a reduced synthesis of undesirable products, like ethanol, in glucose-limited conditions [12, 13]. It also shows a lower basal secretion of proteins when compared to other yeasts, which makes downstream processing easier [13, 14]. Finally, *P. pastoris* can be efficiently cultivated up to high cell densities using fed-batch technology [8], achieving high titers and productivities. For these desirable features, *P. pastoris* has been widely used for the expression of recombinant proteins, reaching grams per liter concentrations in several cases [9, 15–18]. Most remarkably, and as proof of its technical feasibility and adequacy, two recombinant proteins produced in this cell factory have already been approved by the FDA for medical purposes [10, 19].

Despite its growing acceptance and actual successful applications, recombinant protein production in *P. pastoris* can be undermined by several cellular processes, where protein folding and secretion are the most recurrent bottlenecks [14, 20, 21]. In addition, limitations may also be caused by the codon usage of the recombinant protein [22], promoter selection [23], carbon and oxygen availability in the culture [24, 25] and fed-batch operational parameters [26], seriously hampering protein yield, productivity and the economic feasibility of the process.

Industrially, *P. pastoris* is commonly grown in fed-batch cultures in order to maximize the titer and volumetric productivity of a desired compound, often a recombinant protein [27, 28]. This is achieved by adding a culture medium in such a way that the microorganism grows at a desired specific growth rate, which is chosen to maximize the synthesis of the target product and to limit the formation of inhibitory compounds [29]. During this and other cultivation systems, the cells adapt constantly to the changing extracellular environment and to the limited mass transfer conditions observed at high densities [30, 31]. Therefore, it is critical to understand how the cell metabolism interacts with the nutritional and environmental stresses exerted by process conditions to improve bioreactor performance [32]. This is a complex task, however, since the strain’s characteristics and process variables often require significant amounts of time and money for characterization and fine-tuning [12]. Therefore, it is desirable to have a platform to integrate different levels of information from dynamic cultivations of *P. pastoris* that can be used to elaborate rational hypotheses to increase process productivity.

Systems biology offers a quantitative and comprehensive approach to address this task [33]. In particular, Genome-Scale dynamic Flux Balance Analysis (GS-dFBA) [34–36] is a modeling framework that allows the simulation of metabolism during non-stationary (batch or fed-batch) cultures. GS-dFBA models couple the dynamic mass balances of the extracellular environment of the bioreactor with comprehensive mathematical representations of cellular metabolism called Genome Scale Metabolic Models (GSMs). These structures represent the cell’s entire metabolism as a set of underdetermined constrained mass-balances [30, 37, 38]. GSMs have been employed to understand cellular behavior under different environmental conditions, to map over omics data, and to define a metabolic engineering targets [39, 40]. There are currently five published GSMs of *P. pastoris* [41–45] which have been developed to help the strain optimization process with a special emphasis on recombinant protein production. Moreover, one of these models has been successfully employed to improve recombinant protein production in *P. pastoris* [46], validating these frameworks as strain engineering tools for this particular yeast.

GS-dFBA models usually contain several parameters, whose values can be obtained by regression of experimental data. These parameters are used as inputs to obtain flux distributions throughout cultivations, so their values need to be reliable. To ensure this, pre- and post-regression diagnostics have been employed to determine if a certain parameter is supported by the observed data or not [47, 48]. These analyses consist in verifying the model’s capacity to explain the behavior of a system (goodness-of-fit) and the presence of the following parametric limitations: (i) low or no impact on the state variables (sensitivity), (ii) strong correlations with other parameters of the model (identifiability) and (iii) lack of statistical significance (significance). A model is considered robust if it has the capacity to explain different conditions, while containing only sensitive, identifiable and significant parameters.

Here, we present a robust dynamic genome-scale metabolic model of *P. pastoris* in glucose-limited, aerobic batch and fed-batch cultivations. To assemble the dynamic modeling framework, we started by selecting one of the available genome-scale metabolic models [43] and manually curated it to yield realistic flux distributions. Then, we included it in a set of mass balances representing the main compounds present in culture supernatant. Once assembled, the model was calibrated using experimental data from eight batch and three fed-batch cultivations. Next, we employed pre/post regression diagnostics to determine sensitivity, significance and identifiability problems in the model. In order to avoid the aforementioned statistical limitations, problematic parameters were fixed (i.e. removed from the adjustable parameter set) based on the pre/post regression diagnostics, yielding reduced and potentially robust model structures. Potentially robust model structures consisted in the original model formulation with less adjustable parameters. After evaluating these reduced models for each type of cultivation, we chose the one that presented fewer parametric limitations after being re-calibrated with the available data. These reduced models yielded no (or just a few) significance, sensitivity or identifiability problems when calibrating new data and they could predict bioreactor dynamics in conditions like the ones used for their determination. Finally, we carried out simulations to assess the potential of the model to study *P. pastoris* metabolism under industrially relevant conditions, and to select molecular and process engineering strategies to improve recombinant protein production.

## Methods

### Model construction

**Fig. 1:**
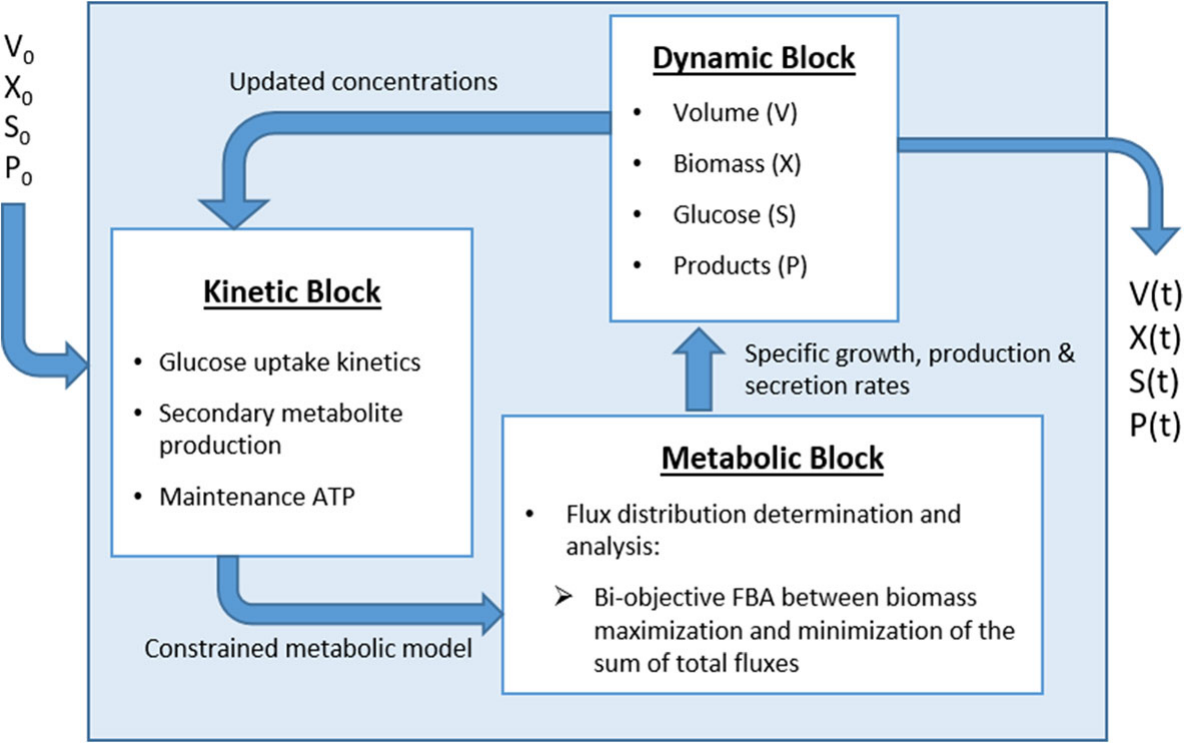
Iterative structure of the model. V refers to culture volume [L], F_IN_ is the feeding policy used in fed-batch cultures, X, S and P are biomass, limiting substrate and Product concentration in [g/L] respectively

The structure of the model was based on an existing dFBA framework developed by Sanchez et al. for *S. cerevisiae* [48], which divides the fermentation time into short integration periods where a metabolic steady state could be assumed [35, 49]. The model considers the evolution of seven state variables throughout batch and fed-batch glucose-limited aerobic cultivations: culture volume as well as the concentrations of glucose, biomass, ethanol, arabitol, citrate and pyruvate. It consists of three linked blocks that are solved iteratively; (i) the kinetic block, (ii) the metabolic block and (iii) the dynamic block (Fig. 1). First, the initial conditions of the system enter into the kinetic block to determine the specific consumption and production rates of the species involved in the analysis according to kinetic expressions. These rates are included as constraints to the corresponding exchange reactions of the metabolic model. The constrained model is then passed to the metabolic block of the framework, where the flux distribution inside the cell is determined. This procedure includes the calculation of the specific growth rate, which is passed along with the other exchange rates to the dynamic block as consumption and production terms in the mass balances. Here, the concentration of the state variables is updated and then incorporated into the kinetic block for the calculation of instantaneous exchange rates. This cycle iterates throughout the cultivation yielding the culture profile and instantaneous flux distributions that can be saved for further analysis. The model is included in Additional file 1 and its latest version can be found online at https://github.com/fjsaitua/RY-dFBA/tree/master/main%20P_pastoris%20dFBA:

#### Kinetic block

The kinetic block sets the uptake and production rates for all the compounds in the model. First, the glucose uptake rate $\left({v}_G\right)$ is determined using Michaelis-Menten kinetics [50].

1 $$ {v}_G=\frac{v_{G,\  Max}\cdot G}{K_G+ G} $$

Here, G is the glucose concentration in the medium [g/L], ${v}_{G, Max}$ is the maximum glucose uptake rate [mmol/g_DCW_ · h] and ${K}_G$ is the uptake half activity constant of this substrate [g/L]. Once determined, $-{v}_G$ [mmol/g_DCW_ · h] is included as the lower bound of the corresponding exchange reaction in the model since substrate consumption is represented with a negative flux through this reaction.

Then, the lower bounds of the exchange reactions ($lb$) associated with the remaining k compounds ($l{b}_k$) are fixed. We considered ethanol, pyruvate, arabitol and citrate dynamics, besides glucose consumption and biomass formation.

2 $$ l{b}_k = {v}_{P_k}\  k=1\dots 4 $$

These parameters are redefined during the fed-batch phase; therefore, they have two values during this type of cultivation.

Finally, the kinetic block fixes the non-growth associated maintenance ATP (m_ATP_, a flux through the cytosolic ATP hydrolysis reaction in the model), which accounts for the energy drain caused by cellular processes not related with the generation of new cell material, such as osmoregulation, shifts in metabolic pathways, cell motility, etc. [51, 52].

#### Metabolic block

The metabolic block receives a constrained GSM from the kinetic block and solves an optimization problem to determine specific growth rate and the flux distribution in the cell. The GSM consists of a set of *m* metabolites and *n* reactions grouped in a Stoichiometric Matrix, S (m x n), that represents the cell’s entire metabolism. If accumulation of metabolites is neglected, a mass balance can be stated according to equation (3):

3 $$ \begin{array}{l} S\cdot v=0\\ {} s.\  t.\\ {} lb< v< ub\end{array} $$

Where $v$ is a vector of metabolic fluxes in [mmol/g_DCW_ · h], and $lb$ and $u b$ are the lower and upper bounds for each component of the flux vector.

The metabolic block solves a bi-objective Quadratic Programming (QP) problem between maximization of growth rate and minimization of the total absolute sum of fluxes [53], subjected to the constraints imposed by the stoichiometric matrix mentioned above [51]:

4 $$ \begin{array}{l} Min\kern0.5em \alpha \cdot {\displaystyle \sum_{i=1}^n}{v}_i^2-\left(1-\alpha \right)\cdot \mu \hfill \\ {} s. t.\hfill \\ {} S\cdot v=0\hfill \\ {} l{b}_i\le {v}_i\le u{b}_i\kern0.5em  i=1\dots n\hfill \end{array} $$

In this formulation, α, the suboptimal growth coefficient, is an adjustable parameter from the model used to modulate the importance of the two – biologically relevant – competing objectives [48, 52, 54]. In our analysis, “optimal growth” occurs when the objective function of the cell is biomass maximization (α = 0). However, when α > 0, the calculated growth rate is lower than the theoretical maximum derived from biomass maximization, at the same glucose uptake rate. In this sense α is considered as a “suboptimal growth coefficient”; it is worthy to note that we do not refer to the optimality of the flux distribution vector, which is actually optimal, given the convexity of the problem in the metabolic block (Equation 4 - See Additional file 2 for details).

The minimization of total fluxes adds a quadratic term to the objective function, which has the practical benefit of eliminating Type III pathways [55] from the flux distribution, which arise from the multiplicity of solutions of a LP problem. These pathways appear as high fluxes (often taking the value of the upper bound of a particular flux) through closed cycles of reactions. This misleads pathway analysis because despite the mass balance around each participating metabolite is satisfied, the fluxes are thermodynamically infeasible [55]. The use of Quadratic Programing makes pathway analysis easier since these large cycling fluxes undermine the minimization of the total fluxes term in the objective function (Equation 4), so they will be forced to a minimum by the optimization software and the flux distribution will be “cleaned” from these unrealistic fluxes. This is especially significant in large networks because these cycles are more recurrent.

In this study, we employed a curated version of the iPP668 model developed by Chung and collaborators [43], called iFS670 (Additional files 3 and 4). In this updated version, we incorporated the arabitol biosynthesis pathway and the stoichiometric reactions for the production of three recombinant proteins (FAB fragment, Human Serum Albumin and Thaumatin). The arabitol synthesis pathway was included because it was a major compound in the culture supernatant of our experiments. Moreover, the reversibility of cytosolic reactions involving redox cofactors and mitochondrial symporters was checked according to Pereira et al. [56] in order to obtain a more realistic flux distribution through the central metabolism. This was done because the initial flux distributions obtained with the un-modified iFS670 model presented the exact same problems as the iMM904 model of *Saccharomyces cerevi*siae on Pereira’s work, suggesting that the central metabolism structure of the iPP668 model was based upon the aforementioned *S. cerevisiae* model. These problems were caused by: (i) the lack of a flux through the oxidative branch of the Pentose Phosphate Pathway; (ii) the presence of a flux of a cytosolic NAPDH dependent isocitrate dehydrogenase (which was the responsible of producing cytosolic NADPH); (iii) an unrealistic flux through mitochondrial symporters; and (iv) almost no mitochondrial formation of α-ketoglutarate. These model limitations are inconsistent with previous *P. pastoris* fluxomic studies in glucose-limited aerobic conditions [24, 57, 58].

FBA problems were solved using the Constraint-Based Reconstruction and Analysis (COBRA) toolbox [59, 60], which employs the programming library libSBML [61] and the SBML toolbox [62]. Finally, we used Gurobi 6.0.2 as an optimization solver.

#### Dynamic block

The dynamic block consists of a set of ordinary differential equations (ODEs) that account for the volume change of the culture and the mass balances of biomass and the species considered by the model:

5 $$ \frac{dV}{dt}= F(t)- S R $$

6 $$ \frac{d\left( V\cdotp X\right)}{ d t}=\mu \cdot \left( V\cdotp X\right)- S R\cdot X $$

7 $$ \frac{d\left( V\cdotp G\right)}{ d t}= F(t)\cdot {G}_F-{v}_G\cdot M{W}_G\cdot \left( V\cdotp X\right) - S R\cdot G $$

8 $$ \frac{d\left( V\cdotp {P}_k\right)}{ d t}={v}_{P_k}\cdotp M{W}_{P_k}\cdot \left( V\cdotp X\right)- S R\cdot {P}_k $$

Where $V$ is volume [L], $t$ is time [h], $F(t)$ is the feed function for the fed-batch phase in [L/h]. $S R$ is a constant sampling rate [L/h] determined from each cultivation to emulate the remaining volume of the culture considering sampling, since this value is used for the calculation of the feeding profile during the feed phase. During the batch phase of the fed-batch cultures, we collected between 15 and 20% of the reactor volume in samples. For batch cultivations, $F(t)$ was eliminated from the mass balances. $X$ is the biomass concentration [g/L], μ is the specific growth rate [h^−1^] (obtained from equation 4), G is the extracellular concentration glucose [g/L], G_F_ is the feed’s glucose concentration [g/L], P_K_ is the k-th extracellular product concentration in [g/L], ${v}_{P_k}$ is the corresponding production rate [mmol/g_DCW_ · h] and MW accounts for the corresponding molecular weight [g/mmol].

The set of equations was solved in Matlab 2013a (Mathworks, USA) using the solvers ode113 and ode15s for batch and fed-batch cultures respectively.

#### Model parameters

The lower, upper and initial values of the parameters of the model used in all the calibrations are presented Table 1. The lower and upper bounds of ${v}_{G, max}$, K_s_, and m_ATP_ were chosen according to literature [29, 43, 63] while the rest of the bounds were selected to ensure that the algorithm had enough search space. To do this, the upper bounds of the rest of the parameters were set at higher values than the observed experimental rates, also taking into account reported values [24, 57, 58]. In addition, initial estimated parameter values were chosen to attain a feasible simulation.

**Table 1:** Parameters of the model

Symbol	Name	Units	LB	Initial value	UB
*v*_*G*,*max*_	Maximum glucose uptake rate	*mmol*/*g*_*DCW*_ *·h*	0	2.5	10
*K*_*G*_	Half saturation constant for glucose uptake	*g*/*L*	0	10^−4^	10^−3^
*v*_*EtOH*,*B*_	Ethanol minimum secretion rate (batch)	*mmol*/*g*_*DCW*_ *·h*	0	0.5	3
*v*_*Pyr*,*B*_	Pyruvate minimum secretion rate (batch)	*mmol*/*g*_*DCW*_ *·h*	0	0.1	2
*v*_*Arab*,*B*_	Arabitol minimum secretion rate (batch)	*mmol*/*g*_*DCW*_ *·h*	0	0.2	2
*v*_*Cit*,*B*_	Citrate minimum consumption rate (batch)	*mmol*/*g*_*DCW*_ *·h*	0	0	2
*v*_*EtOH*,*FB*_	Ethanol minimum consumption rate (fed-batch)	*mmol*/*g*_*DCW*_ *·h*	0	0	2
*v*_*Pyr*,*FB*_	Pyruvate minimum consumption rate (fed-batch)	*mmol*/*g*_*DCW*_ *·h*	0	0	2
*v*_*Arab*,*FB*_	Arabitol minimum consumption rate (fed-batch)	*mmol*/*g*_*DCW*_ *·h*	0	0	2
*v*_*Cit*,*FB*_	Citrate minimum consumption rate (fed-batch)	*mmol*/*g*_*DCW*_ *·h*	0	0	2
*α*_*B*_	Sub-optimal growth coefficient (batch)	[−]	0	0	10^−3^
*α*_*FB*_	Sub-optimal growth coefficient (fed-batch)	[−]	0	0	10^−3^
*m*_*ATP*_	Non-growth associated ATP	*mmol*/*g*_*DCW*_ *·h*	0	2	10
*T*_*Fed*_	Time when secondary metabolite consumption starts in fed-batch cultures	*h*	20	25	32

### Model calibration with experimental data

#### Strains

Four *P. pastoris* strains were employed in this study: a parental GS115 strain (Invitrogen) and three recombinant strains constructed according to the instructions of the manufacturers harboring respectively one, five and eight copies of the gene encoding for the sweet protein thaumatin. Even though the strains were transformed, thaumatin was not detected at concentrations higher than 100 μg/L in the cultivations. Therefore, due to its small contribution to the overall mass balance, thaumatin production was left out of the analysis and none of the parameters of the model were associated with it. Nevertheless, a mass balance for a recombinant protein can be easily added to the framework.

#### Experiments

The batch model was calibrated with aerobic glucose limited cultivations of the four strains available; each cultivation was performed twice. On the other hand, the fed-batch model was calibrated with data from three cultures of the strain with one copy the recombinant gene, under the same environmental conditions of the batch cultivations.

#### Cultivation conditions

Each batch or fed-batch culture started from a 2 [mL] cryotube of the corresponding strain kept at −80 °C. A pre-culture was grown overnight at 30 °C in shake flasks with 50 [mL] of the inoculum medium. After reaching 1 OD_600_, the whole broth was added to 450 [mL] of fresh medium to reach an initial volume of 500 [mL] in 1 L bioreactors. Culture conditions were kept at 30 °C and pH = 6.0. Dissolved Oxygen was maintained above 40% saturation during all the cultivation period. Aerobiosis was achieved by a triple split-range control action, including agitation (200–800 [RPM]), air flow (0.25–1.0 [L/min]) and pure oxygen flow (0–1.0 [L/min]) [64]. pH was controlled using phosphoric acid 20% [v/v] and sodium hydroxide 20% [v/v]. The temperature was controlled with a mixture of hot and cold water, using the glass jacket of the reactors. Lastly, foam was controlled manually using silicone antifoam 10% [v/v]. Glucose starvation was detected when a sudden decrease of the CO_2_ composition in the off-gas occurred, and it was confirmed each time using Benedict’s reagent. For fed-batch experiments, the feed F(t) was designed to track a variable growth rate for a predefined time. This feed can be calculated from the reactor’s glucose and biomass mass balances, as detailed in the literature [65]:

9 $$ F(t)=\frac{\mu_{set(t)}}{G_F\cdot {Y}_{SX}}\cdot {V}_i{X}_i\cdot \exp \left({\displaystyle {\int}_{t_i}^t{\mu}_{set}(t) dt}\right) $$

with G_F_ the glucose feed concentration [g/L], Y_SX_ the experimental glucose-biomass yield [g_DCW_/g] calculated using the genome-scale model, t_i_ the time at which the feed started for a given cultivation [h], V_i_ and X_i_ the volume [L] and biomass [g/L] values at t_i_, respectively, and μ_SET_(t) is the time-dependent user-defined growth rate at which the fed-batch culture is grown. The latter was defined as follows:

10 $$ {\mu}_{set(t)}=\left({\mu}_{max}-{\mu}_{min}\right)\cdot {e}^{- Ct}+{\mu}_{min} $$

Where μ_MAX_ = 0.1 [1/h], μ_MIN_ = 0.07 [1/h] and C = 0.07 [1/h]. Therefore, μ_SET_(t) decays exponentially from 0.1 to 0.07 [1/h], which has been found to increase (in contrast to constant growth rates in the feed phase) the final biomass concentration in fed-batch cultivations of *E. coli* and *S. cerevisiae* performed in our laboratory [66].

#### Culture media

The culture media employed in these studies were based on Tolner et al. [67]. **Inoculum**: Glucose 10 [g/L], (NH_4_)_2_SO_4_ 1.8 [g/L], MgSO_4_ · 7H2O 2.3 [g/L], K_2_SO_4_ 2.9 [g/L], trace elements solution 0.8 [ml/L], histidine 0.08 [g/L], sodium hexametaphosphate 5 [g/L] and biotin 0.32 [mg/L]. **Batch cultures:** Glucose 50 [g/L], (NH_4_)_2_SO_4_ 9 [g/L], MgSO_4_ · 7H_2_O 11.7 [g/L], K_2_SO_4_ 14.7 [g/L], trace elements solution 4 [ml/L], histidine 0.4 [g/L], sodium hexametaphosphate 25.1 [g/L] and biotin 1.6 [mg/L] and sodium hydroxide NaOH 1 [g/L]. **Feeding medium:** Glucose 500 [g/L], MgSO_4_ · 7H2O 9 [g/L], trace solution 12.5 [g/L], histidine 4 [g/L] and biotin 0.1 [g/L]. Sodium hydroxide was added to all the media until a pH of 6 was reached.

#### Analytical procedures

##### Sampling and biomass determination

Samples of ~6 mL were periodically collected (every 2–3 h) from all fermentations. Biomass was measured by optical density (OD) at 600 nm using an UV-160 UV-visible spectrophotometer (Shimadzu, Japan). Biomass concentration was determined using the linear relationship: 1 OD_600_ = 0.72 [g/L] using the methodology from [68]. Then, samples were centrifuged at 10.000 rpm for 3 min and the supernatant stored at −80 °C for further analysis.

##### Extracellular metabolite concentration analyses

Glucose, ethanol, arabitol, citrate and pyruvate extracellular concentrations were quantified in duplicate by High-Performance Liquid Chromatography (HPLC), as detailed in Sánchez et al. [48], with the exception of the working temperature of the Anion-Exchange Column (Bio-Rad, USA), which was lowered from 55 °C to 35 °C for better resolution.

##### Objective Function

For model calibration, we minimized the sum of square errors between the experimental data (Additional files 5 and 6) and the simulation output by searching the parameter space, with the enhanced scatter search algorithm (eSS) [69], which has been successfully used to solve complex bioprocess optimization problems [70–72]. The objective function J used in the minimization was normalized by the maximum corresponding measured variable to give all data a similar weight:

11 $$ J=\underset{\theta}{ \min }{\displaystyle \sum_{i=1}^m}{\displaystyle \sum_{j=1}^n}{\left(\frac{X_{i j}^{m od}-{X}_{i j}^{exp}}{\underset{j}{ \max}\left({X}_{i j}^{exp\ }\right)}\right)}^2 $$

With θ representing the parameter space, m the number of measured variables, n the number of measurements per variable, X_ij_^mod^ the dFBA output of variable i and measurement j, X_ij_^exp^ the corresponding experimental value and $\underset{j}{ \max}\left({X}_{ij}^{exp\;}\right)$ the maximum value measured for variable i.

### Pre/Post regression analysis

Once the initial calibration of the model was completed, statistical tests were performed in order to determine if the initial model formulation had sensitivity, identifiability or significance problems [47].

Sensitivity corresponds to the impact that model parameters have on the state variables or process output. The relative sensitivity of parameter k on the state variable i (g_ik_) was calculated according to the following formula

12 $$ {g}_{i k}\left( t,{\theta}_k\right)=\frac{\theta_k}{X_i(t)}\cdot \frac{d{ X}_i(t)}{d{\theta}_k} $$

Where X_i_(t) is the *i*th state variable in time t and θ_k_ is the *k*th parameter. With all g_ik_ values, we formed a sensitivity matrix g(t) for each experimental time, in which the *k*th column denotes the sensitivity of the kth parameter on the state variables. These matrices were averaged to obtain a single normalized score of the sensitivity of parameter k on the state variable i during the cultivation. Furthermore, if the score of each variable was under 0.01 for a given parameter, this parameter was considered insensitive and a candidate to be fixed (or left out of the adjustable parameter set) in the reparametrization stage.

Identifiability refers to the possibility of unambiguously determining the parameter values by fitting a model to experimental data. If parameter identifiability is not properly assessed, misleading parameter values can be obtained after model calibration. To calculate identifiability, we determined the correlation between the columns of the sensitivity matrix using the *corrcoef* function from Matlab, which yielded a correlation coefficient matrix (C). A pair of parameters j and k was considered to be correlated (therefore not-identifiable) if the absolute value of the number at the (j, k) position in the correlation coefficient matrix was higher than 0.95 ($\left(\left|{C}_{jk}\right|\ge 0.95\right)$).

To determine parameter significance, we started by calculating the Fisher Information Matrix (FIM) [73]

13 $$ F I M={\displaystyle \sum_{j=1}^n}{g}_j^T{Q}_j{g}_j $$

Here, g_j_ is the sensitivity matrix for measurement j, n is the number of samples, and Q_j_ is a weighting matrix given by the inverse of the measurement error covariance matrix assuming white and uncorrelated noise. Hence, the variances for each estimated parameter were calculated as in [73, 74]

14 $$ {\sigma}_k^2= F I{M}_{k k}^{-1} $$

which was used to determine the confidence interval (CI) with 5% significance for the kth parameter as follows:

15 $$ C{I}_k=\left[{\widehat{\theta}}_k\kern0.5em \pm \kern0.5em 1.96{\sigma}_k\right] $$

Here, ${\widehat{\theta}}_k$ is the estimated value of the corresponding parameter. Finally, coefficients of confidence (CC) were calculated as follows:

16 $$ C{C}_k=\frac{\Delta \left( C{I}_k\right)}{{\widehat{\theta}}_k}=\frac{2\cdot 1.96\sigma}{{\widehat{\theta}}_k} $$

Δ(CI_k_) is the CI’s length. A parameter was not significant if the confidence interval contained zero, i. e. if the absolute value of the CC was equal or larger than 2.

### Reparametrization

A reparametrization procedure called HIPPO [75] (Heuristic Iterative Procedure for Parameter Optimization, http://www.systemsbiology.cl/tools/) was applied to overcome parametric statistical limitations in the model.

First, HIPPO performed sensitivity and identifiability tests on the initial calibration results for each dataset. Then, model parameters were fixed one by one until the non-fixed subset presented none of the statistical limitations. Finally, significance was determined for the remaining parameter set, also called the reduced model structure. If all the remaining parameters were significantly different from zero, the resulting structure is considered to be an a priori robust candidate for cross calibration with the available data.

### Cross calibration of robust structure candidates derived from the reparametrization stage using the available datasets

After reparametrization of the model derived from each dataset, a potentially robust structure was generated. This structure was recalibrated with the rest of the datasets to assess its robustness. It is worthy to note that the parameters left out of the calibration were either fixed according to values reported in literature, assumed to be zero or fixed at the mean value achieved in the calibrations. This was done to avoid assuming a minimum production of compounds in batch cultivations and to ensure model convergence for parameters that had no reported values in literature (Table 2). For example, fixing feed phase consumption rates at zero does not allow consumption of batch by-products and yielded poor fed-batch fittings (data not shown).

**Table 2:** Values at which problematic parameters were fixed in the cross-calibration stage

Parameter	Fixation value	Units	Reference
*v*_*G*,*max*_	6	*mmol*/*g*_*DCW*_ *·h*	[63]
*K*_*G*_	0.0027	*g*/*L*	[63]
*v*_*EtOH*,*B*_	0	*mmol*/*g*_*DCW*_ *·h*	-
*v*_*Pyr*,*B*_	0	*mmol*/*g*_*DCW*_ *·h*	-
*v*_*Arab*,*B*_	0	*mmol*/*g*_*DCW*_ *·h*	-
*v*_*Cit*,*B*_	0	*mmol*/*g*_*DCW*_ *·h*	-
*v*_*EtOH*,*FB*_	1.21	*mmol*/*g*_*DCW*_ *·h*	*
*v*_*Pyr*,*FB*_	0.14	*mmol*/*g*_*DCW*_ *·h*	*
*v*_*Arab*,*FB*_	0.15	*mmol*/*g*_*DCW*_ *·h*	*
*v*_*Cit*,*FB*_	0.008	*mmol*/*g*_*DCW*_ *·h*	*
*α*_*B*_	0	[−]	[85]
*α*_*FB*_	0	[−]	[85]
*m*_*ATP*_	2.18	*mmol*/*g*_*DCW*_ *·h*	[43]
*T*_*Fed*_	22	*h*	*

Parameters marked with ‘-’ in the reference column indicate that no a priori value was assumed for that particular parameter, which is the case for the batch minimum secretion rates. ‘*’ means that the value of a particular parameter was fixed at the mean value achieved in the calibrations, because no information about them could be found in the literature

The reduced modeling structures were evaluated according to four parameters:

I. **Relative difference between calibration objective functions (J**_**DIFF**_ **):**
  17 $$ {J}_{DIFF}=\frac{1}{n}\cdot {\displaystyle \sum_{i=1}^n}\frac{J_{i, Reduced}-{J}_{i, Original}}{J_{i, Original}} $$
  Where n corresponds to the number of cultures of each type, ${J}_{i, Original}$ is the calibration objective function (Equation 11) achieved for dataset i using the original model structure and ${J}_{i, Reduced}$ is the calibration objective function achieved in dataset i using a reduced, a priori robust, modeling structure.
II. **Percentage of Significance issues**; refers to the number of times a parameter is found to be non-significant out of the total of significance determinations performed for a structure. For instance, if a model structure had 6 parameters and 8 datasets were used to calibrate it, a total of 48 significance determinations were performed for that particular model.
III. **Percentage of Sensitivity issues;** refers to the number of times one of the estimated parameters shows low or no impact over state variables (average relative sensitivity ≤ 0.01) out of the total sensitivity determinations performed.
IV. **Percentage of Identifiability issues**; corresponds to the number of times a pair of parameters presents a strong correlation (≥0.95), out of the total parameter pairs of a modeling structure. If p is the number of parameters of the model and n is the number of datasets used for its calibration, the total of parameter pairs for which identifiability was determined is:
  18 $$ Total\  pairs=\frac{p\cdot \left( p-1\right)}{2}\cdot n $$

Finally, the modeling structure that presented the lowest J_DIFF_ and fewest statistical limitations was used as a robust structure candidate for the corresponding type of culture.

### Robustness check of the chosen modeling structure

Once a candidate for a robust structure was determined for the batch and fed-batch configurations, we tested its robustness (absence of parametric problems) by calibrating it with new experimental data. For the batch model, we employed fermentation data from *P. pastoris* GS115 strain grown with 40 [g/L] of glucose as carbon source at T° = 25 °C and pH = 6. The robustness of the fed-batch model was assessed with a glucose-limited cultivation consisting of a 60 [g/L] glucose batch phase and an exponential feed using 500 [g/L] of glucose. The medium was added in the feeding phase in order to achieve an exponentially decreasing growth rate from 0.1 to 0.07 [1/h].

### Model validation

Finally, the predicting capability of the model was evaluated for conditions similar to the ones used in the initial calibrations (training set).

The robust batch model was first calibrated with the two cultivations of the strain harboring one copy of the thaumatin gene, obtaining a characteristic parameter set for that strain. Then, these parameters were used to predict the course of a different batch cultivation performed in the same conditions (30 °C and pH 6).

This procedure was also applied for the fed-batch model. Here, the bioreactor dynamics was simulated using the parameters obtained in the best calibration within the training dataset (the one in which the calibration objective function was minimal compared to the rest of the calibrations) using the robust modeling structure obtained previously. This prediction was compared with experimental data of a different fed-batch cultivation.

### Goodness of fit

For both the robustness check and validation datasets, the goodness of fit was determined by two scores: the mean normalized error (MNE) and the Anderson-Darling test [76]. The MNE quantifies the difference between model simulations and experimental data; the closer the difference is to zero, the better the fit. In addition, the sign of MNE shows whether the model over (+) or underestimates (−) the observed data (equation 19).

19 $$ M N{E}_i=\frac{{\displaystyle {\sum}_{j=1}^n}\left({X}_{i j}^{mod}-{X}_{i j}^{exp}\right)}{n\cdot \underset{j}{ \max}\left({X}_{i j}^{exp\ }\right)} $$

with n the number of time points measured for variable i.

The Anderson-Darling test was used to verify if the residuals between simulations and experimental data $\left({X}_{ij}^{mod}-{X}_{ij}^{exp}\right)$ were normally distributed. If they were, the differences between them can be attributed to measurement noise and not to model inadequacy. The failure of this test by one of the model’s state variables (p-value < 0.05) indicates that a different mathematical relation than the one used in the model may underlie its dynamics. Therefore, the results of this test may be used to confirm or update the kinetic expressions associated with the consumption and production of compounds.

### Simulation

#### Analysis of the metabolic flux distribution during key stages of a dynamic cultivation

After the calibration of the fed-batch model with the dataset used for checking its robustness, we evaluated the central metabolic flux distributions at three different stages of the cultivation: exponential growth during the batch phase (~20 h), ethanol and arabitol consumption during glucose starvation phase (~27.5 h) and controlled growth during the feeding phase (~45 h).

#### Discovery of beneficial knock-out targets for the overproduction of recombinant Human Serum Albumin (HSA)

To show the potential applications of the model, gene targets for the overproduction of the recombinant Human Serum Albumin (HSA) were determined by simulating the growth and protein secretion of single knock-out strains of *P. pastoris* in batch cultivations. To do this, we included in the Metabolic Block a second quadratic programing problem consisting in the Minimization of Metabolic Adjustment (MOMA) algorithm [77], which states that, after a genetic perturbation, the cell will attempt to redistribute its metabolic fluxes as similar as possible to the parental strain. Mathematically, equation 4 of the metabolic block is employed in order to obtain the parental flux distribution ${v}_0$ at a given instant.

20 $$ \begin{array}{l} Min\ \alpha \cdot {\displaystyle \sum_{i=1}^n}{v}_{0, i}^2-\left(1-\alpha \right)\cdot {\mu}_0\hfill \\ {} s. t.\hfill \\ {} S\cdot {v}_0=0\hfill \\ {} l{b}_{0, i}\le {v}_{0, i}\le u{b}_{0, i}\kern1em  i=1\dots n\hfill \end{array} $$

Then, the k reactions associated with gene j are blocked:

21 $$ l{b}_{l, j}= u{b}_{l, j}=0\  l=1\dots k $$

Finally, the MOMA algorithm was applied using the flux distribution of the parental strain ${v}_0$ to calculate the knockout distribution ${v}_{KO}$ as the Euclidean distance between them, considering that the actual model has the corresponding deletion.

22 $$ \begin{array}{cc}\hfill \mathrm{MOMA}:\hfill & \hfill \hfill \\ {}\hfill \hfill & \hfill \begin{array}{l} Min\ {\left({v}_0-{v}_{KO, j}\right)}^2\hfill \\ {} s. t.\hfill \\ {} S\cdot {v}_{KO, j}=0\hfill \\ {} l{b}_i\le {v}_{i, KO, j}\le u{b}_i\kern2em  i=1\dots n\hfill \end{array}\hfill \end{array} $$

**Fig. 2:**
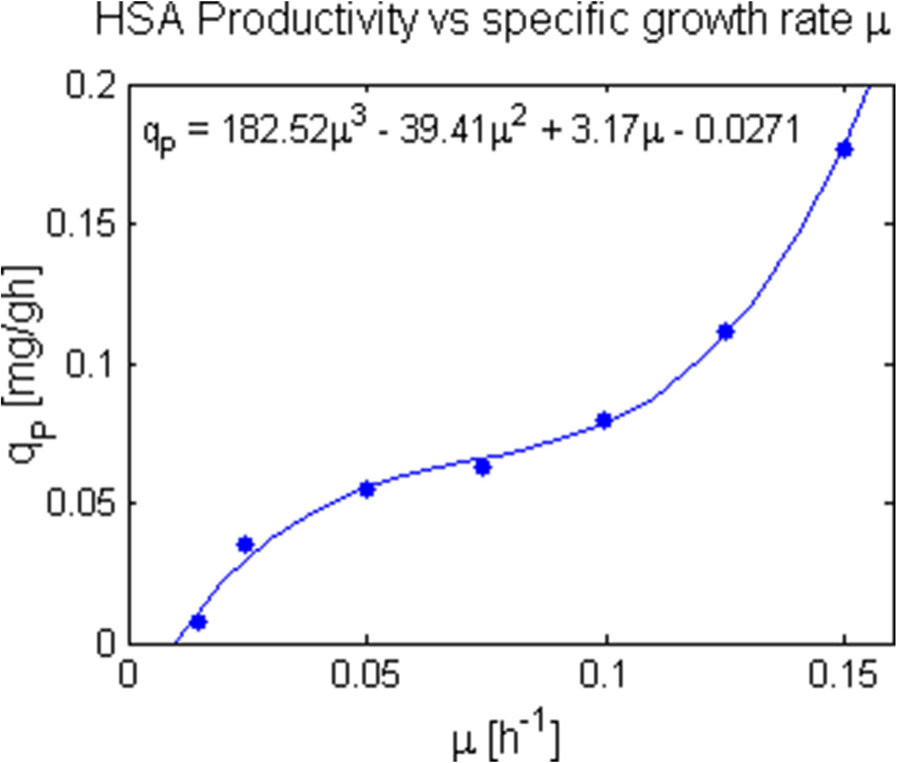
Relation between Human Serum Albumin specific production rate (q_P_) and growth rate (μ) in glucose limited chemostats, taken from Rebnegger et al. [78]. This relation was included to simulate the specific protein productivity for a given growth rate, allowing the assessment of the impact of different feeding profiles on process productivity

The hypothetical parental strain was characterized using the parameters obtained above plus the growth rate dependent specific HSA productivity (q_P_) of *P. pastoris* strain SMD1168H grown on glucose, as reported by Rebnegger et al. [78], (Fig. 2). In each iteration of the model, the minimum HSA production was fixed according to this relationship, which was fitted with a third degree polynomial. Other kinetic expressions could be employed to represent the q_P_ vs μ relationship, depending on the strain and protein being produced [26].

We simulated one batch cultivation for each gene in the model and compared their final protein and biomass concentrations with those of the parental strain. The candidates that reached a higher HSA concentration than the parental strain were manually analyzed and some of them were proposed as candidates to improve HSA production. It is important to mention that we used a set of parameters derived in this study to characterize the growth kinetics of the HSA producing strain used in the simulations. Therefore, the predictions derived from this work should be assessed carefully and considered only as an example of the applicability of our modeling framework.

#### Evaluation of different feeding policies *in silico* to improve recombinant protein production considering specific information about the strain and process setup

Simulations were run using the parameters obtained in the calibration used for intracellular flux analysis and adding the q_P_ vs μ relation for HSA biosynthesis in the mass balances. The process limitations (based on our setup) were a maximum reactor volume of 1 L, and a maximum oxygen transfer rate of 10.9 [g/L · h]. If any of these limits were violated by either the feeding rate of medium or the oxygen uptake rate (extracted from the model), the integration stopped.

We assessed 13 exponential feeding policies. Five of them maintained a constant growth rate during the feeding phase and the rest considered a decreasing growth rate throughout the culture (Additional file 7). After the simulation, we ranked the strategies according to the volumetric productivity of recombinant HSA and chose the best one as a cultivation strategy that could potentially improve bioreactor performance.

## Results and discussion

The batch and fed-batch models were developed in four steps: (i) determination of initial parametric problems, (ii) reparametrization and cross calibration, (iii) robustness evaluation and (iv) validation of predictive potential under the studied conditions.

Once the models were developed, three applications were proposed to improve recombinant |protein production using Human Serum Albumin as a case study.

### Initial parametric problems

#### Batch model

The initial structure of the batch model comprised eight parameters (Table 3). The model was able to successfully accommodate different cellular dynamics from eight glucose-limited aerobic cultivations. In these calibrations, several statistical parametric limitations were found (Additional file 8). m_ATP_ was the parameter that presented the strongest correlation with other parameters, such as maximum specific glucose uptake rate $\left({v}_{G, Max}\right)$, ethanol and arabitol specific secretion rates $\Big({v}_{EtOH, B}\; and\;{v}_{Arab, B}$), and with the sub-optimal growth coefficient (${\alpha}_B\Big)$. This might result from the fact that a change in m_ATP_ directly impacts the ATP-producing pathways in the metabolic model, affecting the biomass and product yields, which are also influenced by other parameters of the model. In addition, the glucose uptake saturation constant K_G_ was the only parameter with frequent sensitivity and significance problems, making it a potential candidate to be left out of the adjustable parameter set.

**Table 3:** Potential Robust Structures Tested in the Cross-Calibration Stage for the batch model

Structure	Parameters included
Original	*v*_*G*, *Max*_, *K*_*G*_ *v*_*EtOH*,*B*_, *v*_*Pyr*,*B*_, *v*_*Arab*,*B*_, *v*_*Cit*,*B*_, *m*_*ATP*_ *and α*_*B*_
1	*v*_*G*,*Max*_, *v*_*EtOH*,*B*_, *v*_*Pyr*,*B*_, *v*_*Arab*,*B*_, *v*_*Cit*,*B*_ *and α*_*B*_
2	*v*_*G*, *Max*_, *v*_*Cit*,*B*_ *and α*_*B*_
3	*K*_*G*_, *v*_*EtOH*,*B*_, *v*_*Pyr*,*B*_, *v*_*Arab*,*B*_ *and v*_*Cit*,*B*_
4	*v*_*EtOH*,*B*_ *and v*_*Cit*,*B*_
5	*v*_*G*,*Max*_, *v*_*Pyr*,*B*_, *v*_*Arab*,*B*_
6	*v*_*G*,*Max*_, *v*_*EtOH*,*B*_, *v*_*Pyr*,*B*_, *v*_*Arab*,*B*_, *v*_*Cit*,*B*_
7	*v*_*G*,*Max*_, *K*_*G*_, *v*_*EtOH*,*B*_, *v*_*Pyr*,*B*_, *v*_*Cit*,*B*_
8	*K*_*G*_, *v*_*Pyr*,*B*_, *v*_*Arab*,*B*_, *α*_*B*_ *and m*_*ATP*_

Each one of these structures was derived using HIPPO after model calibration using each dataset

#### Fed batch model

Data from three aerobic, glucose-limited fed-batch cultivations was successfully calibrated with the initial model of fourteen parameters. As in the batch model, several statistical parametric limitations arose (Additional file 8). The most frequent correlation (in two out of the three calibrations) was between ${v}_{G, Max}$ and the ${v}_{EtOH, B}$ during the batch phase. Also, ${v}_{EtOH, B}$ and ${v}_{Arab, FB}$ showed 5 and 6 strong correlations with other parameters of the model, respectively.

Finally, the citrate minimum secretion rate during the fed-batch phase and the suboptimal growth during the feeding phase (${\alpha}_{FB}$) were the parameters that presented more sensitivity and significance limitations.

### Reparametrization and cross calibration

After model calibration and the subsequent determination of the parametric problems for each dataset, the non-relevant parameters were fixed (left out of the adjustable set) using HIPPO [75] to achieve robust modeling structures.

#### Batch model

The reduced batch models derived from the initial calibrations (Table 3) were recalibrated with the available data (eight batch cultivations) to determine if they could reproduce *P. pastoris* behavior appropriately. The persistence of parametric problems in the reduced models was compared to the original model.

Structures 1 and 6 were the only parameter sets whose fitting capabilities were similar to the original eight parameters model (Table 4), showing the importance of including the specific uptake and production rates of the compounds considered in the model. On the contrary, ${m}_{ATP}$ and ${K}_G$ were left out of these structures because of the frequent identifiability and sensitivity associated problems.

**Table 4:**
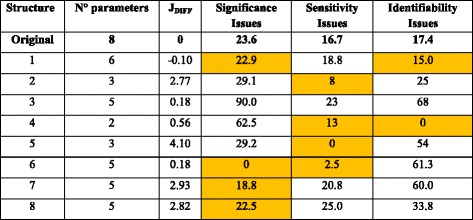
Batch cross calibration summary

Structures that reduced de frequency of parametric problems with respect to the original model are highlighted

Structure 6 lacks the sub-optimal growth parameter ${\alpha}_B$, which forces the solution of a linear programming (LP) problem of specific growth rate maximization in the metabolic block. This is because this parameter was assumed to be zero if it was left out of the adjustable parameter set (Table 2), which eliminates the total flux minimization term from the objective function. This structure showed a significant increase in significance and sensitivity compared to the original model; however, identifiability was a major problem (Table 4). Probably, the multiple solutions associated with an underdetermined LP problem may hamper the possibility to unambiguously infer parameter values from the data.

Therefore, due to the recurrent identifiability issues found in Structure 6, it was preferable to apply Structure 1 to fit a different dataset to check its robustness in aerobic, glucose-limited batch cultures of *P. pastoris*.

#### Fed-batch model

In the fed-batch model, three potentially robust model structures were found after its calibration with three datasets (Table 5).

**Table 5:** Potential robust structures for a fed-batch model

Structure	Parameters included
Original	*v*_*G*,*Max*_, *K*_*G*_, *v*_*EtOH*,*B*_, *v*_*Pyr*,*B*_, *v*_*Arab*,*B*_, *v*_*Cit*,*B*_, *v*_*EtOH*,*FB*_, *v*_*Pyr*,*FB*_, *v*_*Arab*,*FB*_, *v*_*Cit*,*FB*_, *α*_*B*_, *α*_*FB*_, *m*_*ATP*_, *T*_*Cons*_
1	*v*_*G*,*Max*_, *K*_*G*_, *v*_*Pyr*,*B*_, *v*_*Cit*,*B*_, *v*_*EtOH*,*FB*_, *v*_*Pyr*,*FB*_, *v*_*Arab*,*FB*_, *v*_*Cit*,*FB*_, *α*_*B*_, *m*_*ATP*_, *T*_*Cons*_
2	*K*_*G*_, *v*_*EtOH*,*B*_, *v*_*Pyr*,*B*_, *v*_*Arab*,*B*_, *v*_*Cit*, *B*_, *v*_*EtOH*,*FB*_, *v*_*Pyr*,*FB*_, *α*_*B*_, *m*_*ATP*_
3	*v*_*G*,*Max*_, *K*_*G*_, *v*_*Pyr*,*B*_, *v*_*Arab*,*B*_, *v*_*Cit*,*B*_, *v*_*Pyr*,*FB*_ *α*_*B*_, *α*_*FB*_, *m*_*ATP*_, *T*_*Cons*_

**Fig. 3:**
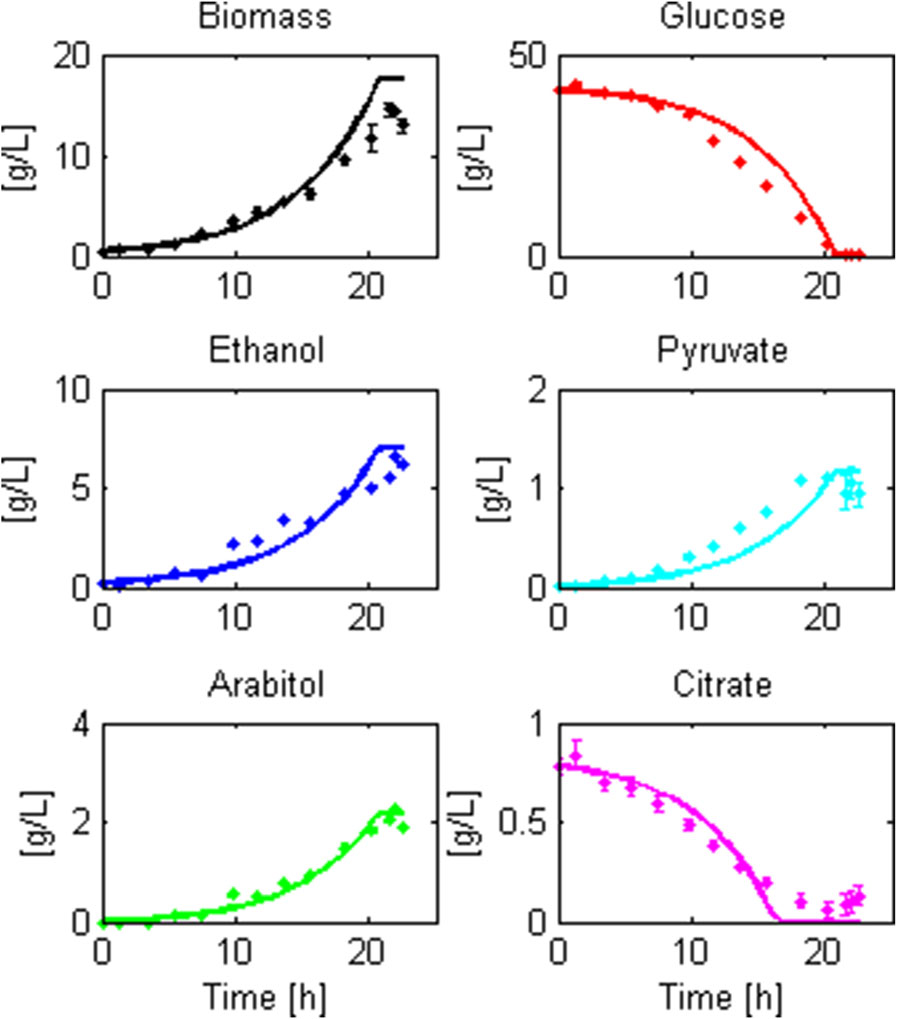
Robustness check of Structure 1 as modeling framework for aerobic, glucose-limited batch cultures of *Pichia pastoris*. The figure shows the capacity of the reduced model structure to be calibrated with new data despite having fewer parameters than the original model structure (6 instead of 8 parameters). Points with whiskers represent experimental data and continuous lines correspond to the model approximation

All the candidate structures considered the following parameters: ${K}_G$, ${v}_{Pyr, B}$, ${v}_{Cit, B}$, ${\alpha}_B$, ${v}_{Pyr, FB}$ and ${m}_{ATP}$. Contrary to the batch model, ${K}_G$ plays an important role in this cultivation system. This parameter, which usually lies in the micromolar range [79], can directly modulate substrate uptake under glucose-limited conditions. Therefore, when glucose concentration is close to zero (like in the feeding phase), slight variations in the value of ${K}_G$ can change glucose uptake significantly, which has a direct impact in the specific growth rate. Also, ${m}_{ATP}$ appears to have a relevant role since it might act as an energy sink when the glucose from the batch phase is depleted. Here, secondary product consumption occurs with a slower or null biomass formation prior to the addition of glucose (Fig. 3 in Additional file 8). This indicates that the substrates were consumed to maintain basic cellular functions to survive, instead of being used for cell division.

The three reduced structures improved the initial fittings (lower J_DIFF_) and reduced the frequency of fitting problems observed in the initial model of 14 parameters (Table 6). Among these, Structure 3 performed the best in the cross-calibration in terms of fitting capability compared to the original model. On average, this structure improved in initial calibrations by 25%. It is worthy to note that, even though Structure 3 did not include the minimum production rate of ethanol during the batch phase, it could adequately reproduce the profiles of this compound by adjusting the objective function and the maintenance ATP. Finally, we chose to apply Structure 3 to fit new fed-batch data to check its robustness for modeling glucose-limited aerobic fed-batch cultivations of *P. pastoris*.

**Table 6:**
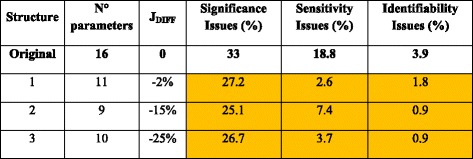
Summary of the cross calibration of the fed-batch datasets

Structures that reduced de frequency of parametric problems with respect to the original model are highlighted

### Robustness check

#### Batch model

On this new dataset, Structure 1 showed a good fit to the data and did not yield identifiability nor significance problems. However, ${v}_{G, Max}$ had no impact on the state variables. Therefore, after the initial calibration (data not shown), we fixed this parameter at 6 [mmol/g_DCW_h] [63]. Figure 3 illustrates the model fit and Table 7 presents the parameter values with their 95% confidence intervals achieved in the second calibration, which also had no identifiability, significance or sensitivity limitations. This calibration also yielded mean normalized errors close to zero and normally distributed residuals for all the state variables except for glucose (Additional file 9).

**Table 7:** Parameter values achieved in the validation of the batch model structure

Parameter	Value	Units
*v*_*G*,*Max*_	6	mmol/g_DCW_ · h
*v*_*EtOH*,*B*_	1.47 ± 0.07	mmol/g_DCW_ · h
*v*_*Pyr*,*B*_	0.13 ± 0.05	mmol/g_DCW_ · h
*v*_*Arab*,*B*_	0.14 ± 0.06	mmol/g_DCW_ · h
*v*_*Cit*,*B*_	0.09 ± 0.04	mmol/g_DCW_ · h
*α*_*B*_	4.1 ± 0.9 · 10^−4^	[−]

Values of the parameters are presented together with their 95% confidence intervals. In this calibration, *v*_*G*,*Max*_ was fixed at a known value to avoid sensitivity issues. Finally, the calibration yielded no parametric problems

Despite the sensitivity problem associated with ${v}_{G, Max}$ for this particular dataset, we included this parameter in the proposed robust modeling structure. This is because for some calibrations, e.g. the batch cultivations of strains harboring 8 copies of the thaumatin gene, the state variables were very sensitive to this parameter (average sensitivity > 0.7, recall that the sensitivity threshold is 0.01); hence, it should be included to achieve a close fit to the data. Therefore, if this parameter is found insensitive in future calibrations, it could be easily fixed at reported values.

We achieved a robust modeling structure for glucose-limited, aerobic batch cultivations of *Pichia pastoris*, composed of six parameters that estimate specific consumption and production rates of all the species involved in the mass balances. The modeling structure also allows us to determine the specific growth rate by solving a bi-objective optimization problem, which reduces the identifiability issues arising between parameters (comparison between candidate batch model robust structures 1 and 6).

#### Fed-batch model

**Fig. 4:**
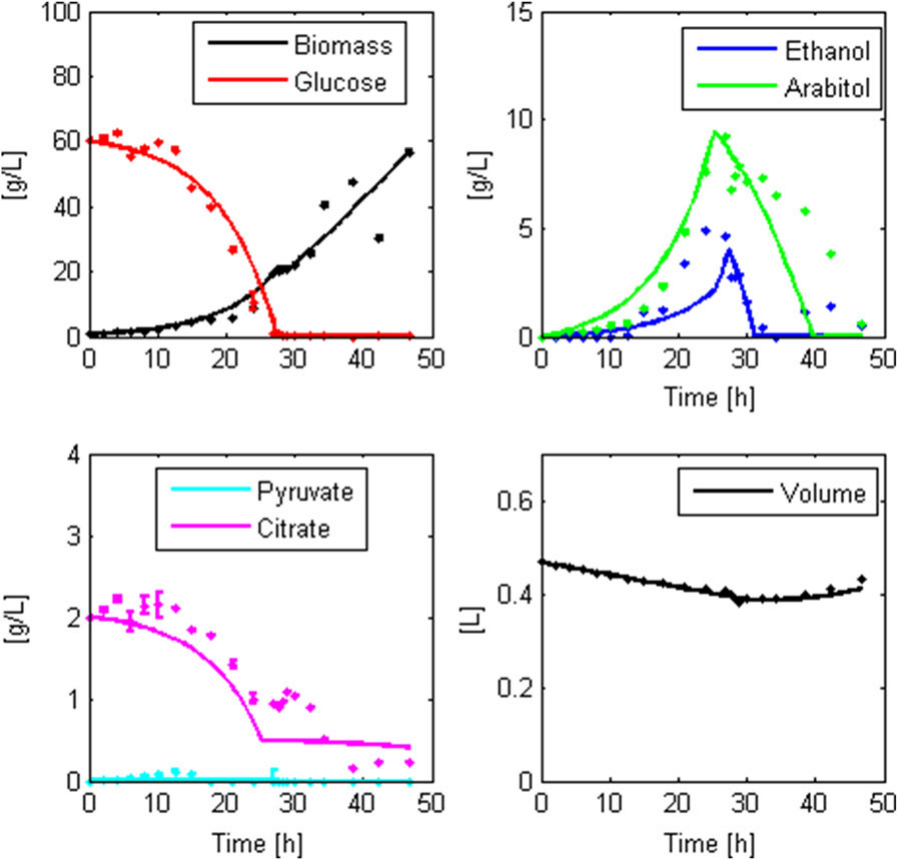
Robustness check of Structure 3 as a modeling framework of aerobic glucose-limited fed-batch cultures of *Pichia pastoris*. The figure shows the capacity of the reduced model structure to be calibrated with new data, despite having fewer parameters than the original model structure (10 instead of 14 parameters). Points with whiskers represent experimental data and continuous lines correspond to the model approximation

Structure 3 shows a good fit to new experimental fed-batch data (Fig. 4) and did not yield identifiability or significance problems (Table 8 and Additional file 9). The profile of some of the state variables still depends on the fixed values assigned. For example, arabitol was consumed at a slower rate than the profile observed in the experiment because the parameter representing this consumption (${v}_{Arab, FB}$) was fixed as the mean of the training datasets (not included in the adjustable parameter set). Thus, the model assumed a faster consumption rate than observed in the cultivations. Also, pyruvate was found at such low concentrations that the parameters associated to its production (${v}_{Pyr, B}\; and\;{v}_{Pyr, FB}$) were ignored in this analysis.

**Table 8:** Parameter values achieved in the calibration to check the robustness of the fed-batch model. The confidence interval on the time where the consumption of secondary metabolites started T_CONS_, could not be determined due to the stiffness of the solution caused by a sudden consumption of arabitol and ethanol

Parameter	Value	Units
*v*_*MAX*_	2.09 ± 0.46	mmol/g_DCW_ · h
*K*_*S*_	5.55 · 10^−2^ ± 0.0000004 · 10^−2^	g/L
*v*_*Pyr*,*B*_	0	mmol/g_DCW_ · h
*v*_*Arab*,*B*_	0.42 ± 0.17	mmol/g_DCW_ · h
*v*_*Cit*,*B*_	0.04 ± 0.00	mmol/g_DCW_ · h
*v*_*Pyr*,*FB*_	0	mmol/g_DCW_ · h
*α*_*B*_	2.6 · 10^−4^ ± 0.4 · 10^−4^	[−]
*α*_*FB*_	2.455 · 10^−5^ ± 0.003 · 10^−5^	[−]
*m*_*ATP*_	7.0 ± 1.4	mmol/g_DCW_ · h
*T*_*Cons*_	25.73	H

The chosen model structure showed a strong fitting capacity and a limited occurrence of parametric identifiability, sensitivity and significance problems. Therefore, we selected it as the most robust model structure for fed-batch cultivations of *P. pastoris*.

### Model validation

#### Batch model

**Fig. 5:**
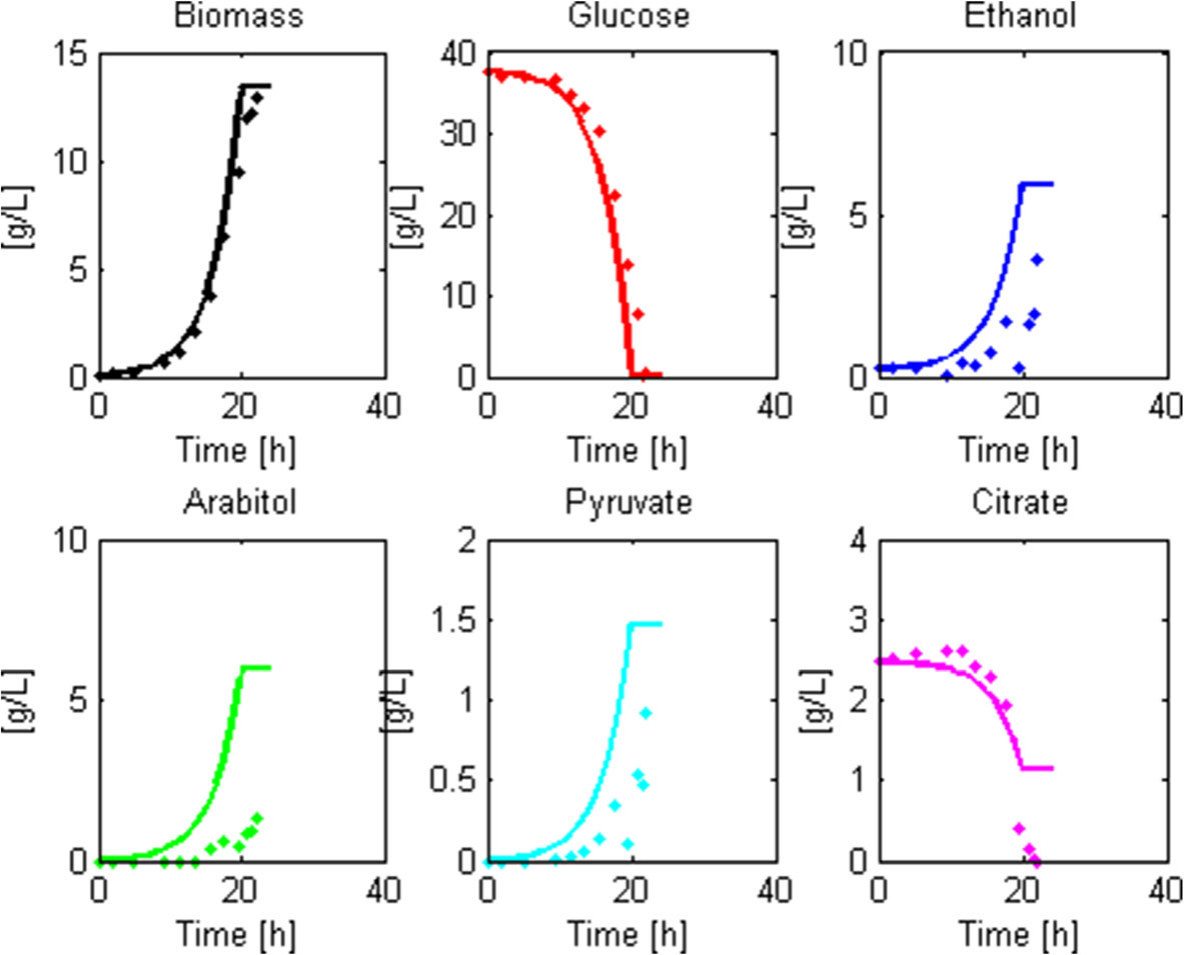
Batch model preliminary validation. This figure shows how well the model predicts the course of a batch cultivation. To do this, we used the derived robust model structure to determine the characteristic parameters of a recombinant strain. Then, we simulated a batch culture (*continuous line*) and compared it with the experimental data (*filled circles*)

The parameters found for the strain harboring one copy of the thaumatin gene were used to predict the dynamics of a different batch cultivation using the same strain (Fig. 5). Biomass and glucose profiles were correctly predicted by the model (MNEs close to zero and p-values of the Anderson-Darling test > 0.05, see Additional file 10). Ethanol, pyruvate, citrate and arabitol dynamics also showed an overall concordance with the data, however the simulated profiles overestimated their final concentrations (see associated MNEs in Additional file 10). These differences occurred probably because in the training datasets the initial concentration of glucose was higher than the one used in the validation experiment (~60 g/L vs. ~40 g/L), which might have increased the formation of secondary products [80]. Therefore, future versions of the model may consider more elaborate kinetic expressions for the secretion of secondary products in order to accurately predict their formation in different circumstances.

#### Fed-batch model

**Fig. 6:**
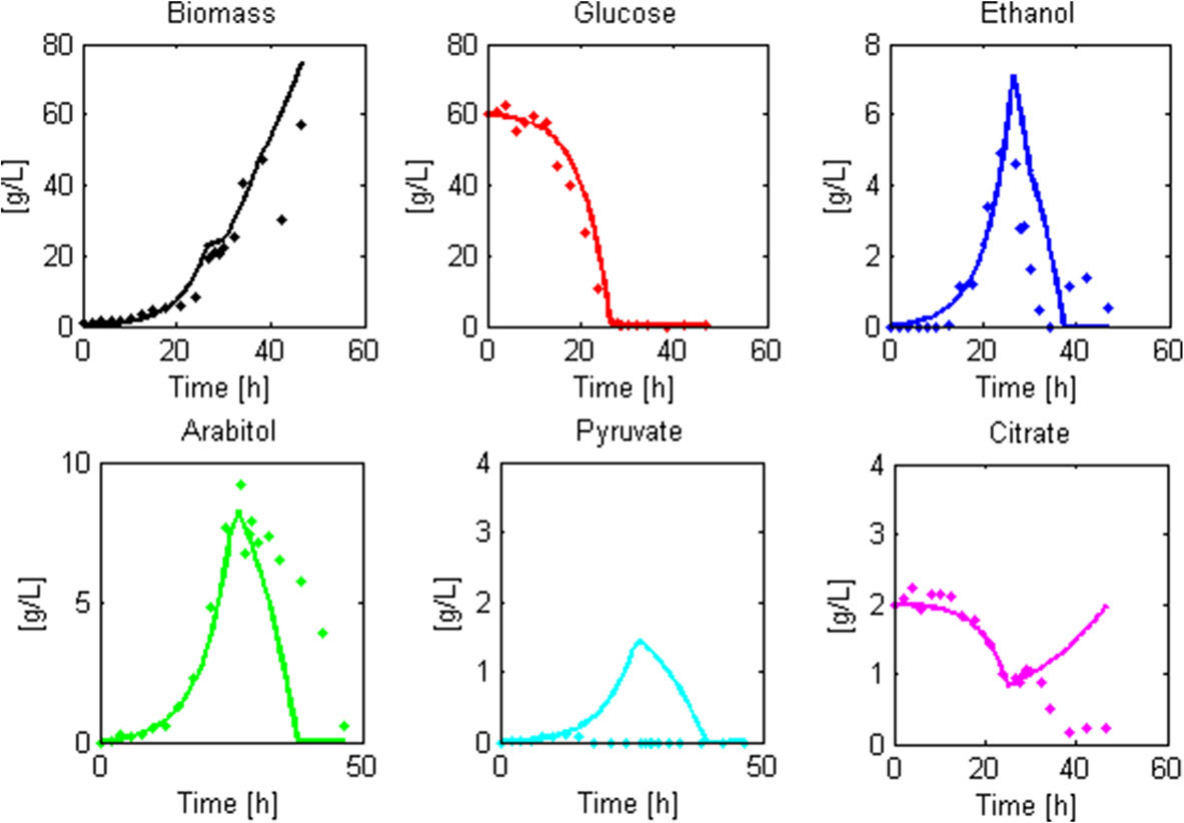
Fed-batch model validation. This figure shows how well the model predicts the course of a fed-batch cultivation. To do this, we used the derived robust model structure to determine the characteristic parameters of a recombinant strain. Then, we simulated a fed-batch culture (*continuous line*) and compared it with the experimental data (*filled circles*)

The prediction of biomass, glucose, ethanol and arabitol concentrations during the culture agreed with experimental data, whereas pyruvate and citrate dynamics were inaccurate (Fig. 6). Specifically, the simulation predicted that pyruvate was generated during the batch phase but experimental data did not show pyruvate production. In the experimental culture we saw that there was no generation of citrate in the feed phase, contrary to what the simulated predicted. These differences arose because in the culture from where the parameters were derived (Fed-batch culture 1, see Additional file 8), pyruvate formation occurred in the batch phase and citrate was formed during the feed phase; therefore, the model assumed that these compounds were generated in the respective phase of the culture. Nevertheless, for the major compounds found in the culture, the model had a low mean normalized error.

### Potential applications of the model

#### Analysis of the metabolic flux distribution at different stages of a dynamic cultivation

**Fig. 7:**
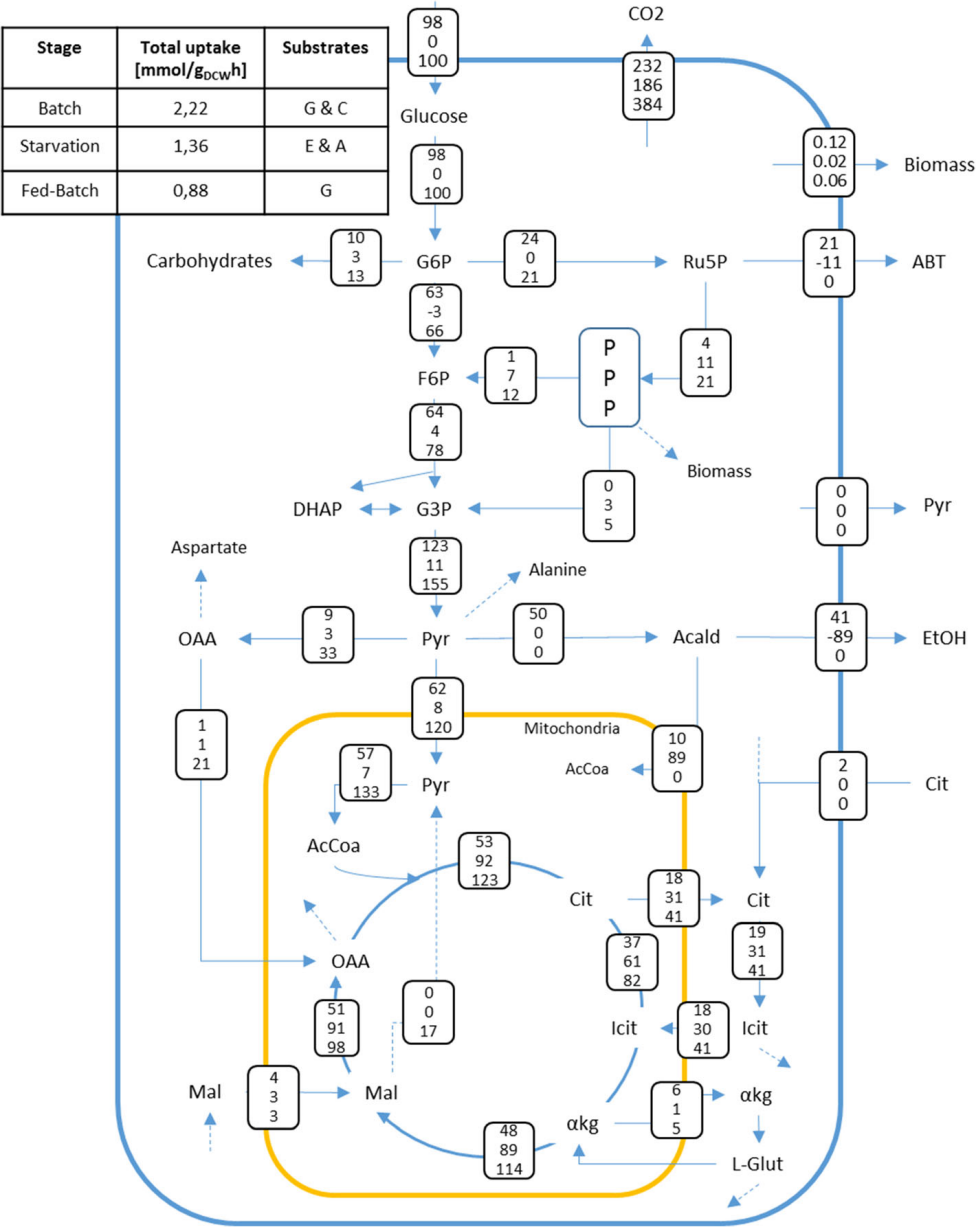
Metabolic flux distribution in the Central metabolism for three different stages of the cultivation. Carbon uptake is detailed in the box of the upper left corner in mmol/g_DCW_h and the fluxes are presented relative to this uptake. In each box between metabolites there are three numbers which correspond, from top to bottom, to the relative flux during batch, starvation and feeding phases. Depending on the time analyzed, the cell consumes Glucose (G), Citrate (C), Arabitol (A) or Ethanol (E). The biomass flux corresponds to the specific growth rate of the cell in h^−1^ and the negative fluxes refer to a change in the reaction directionality. Nomenclature: G6P = Glucose 6 Phosphate, Ru5P = Ribulose 5 Phosphate, ABT = Arabitol, PPP = Non-oxidative phase of the Pentose Phosphate Pathway, F6P = Fructose 6 Phosphate, G3P = Glyceraldehyde 3 Phosphate, DHAP = Dihydroxyacetone Phosphate, Pyr = Pyruvate, OAA = Oxaloacetate, Acald = Acetaldehyde, EtOH = Ethanol, AcCoA = Acetyl Coenzyme A, Cit = Citrate, Icit = Isocitrate, αkg = Alpha-keto glutarate, Mal = Malate and L- glut = Glutamate

Once we confirmed the robustness of the fed-batch model, we analyzed the redistribution of central carbon metabolic fluxes at three different stages of the cultivation (Fig. 7), i.e. exponential growth during the batch phase (~20 h, μ = 0.12 h^−1^); co–consumption of arabitol and ethanol during the glucose starvation phase (~27.5 h, μ = 0.02 h^−1^); and controlled exponential growth during the feeding phase (~45 h, μ = 0.06 h^−1^) (Fig. 7).

During exponential growth in the batch phase, the carbon reaching the glucose-6-phosphate node is split between carbohydrate production (11%), glycolysis (63%) and the oxidative branch of the PPP (24%). Furthermore, the latter is the main source of cytosolic NADPH. Cytosolic ATP is formed by the activity of the ATP synthase and substrate-level phosphorylation (glycolysis and synthesis of arabitol and ethanol) (data not shown). In the iPP618 model, which is the basis of the iFS670, cytosolic NADPH was produced by a NADP dependent isocitrate dehydrogenase, and no flux appeared through the oxidative branch of the PPP. Using the proposals from Pereira et al. [56], the flux through this pathway was restored and overall agreement in directionality to fluxomic studies performed in similar conditions was achieved (Additional file 3).

During the starvation phase, ethanol and arabitol are co-consumed with limited formation of biomass (μ = 0.02 h^−1^). As indicated by the negative fluxes, both compounds are directed towards the TCA cycle in order to synthesize the necessary reducing equivalents to fuel oxidative phosphorylation. The ATP formed in this pathway - ~ 7 mmol/g_DCW_·h -, is mostly employed for maintenance. Even though this m_ATP_ is high compared to other reported values for *P. pastoris* (2.2 – 5 mmol/g_DCW_·h) [43], it is required to account for the fast consumption of both secondary metabolites under limited cellular growth. The use of a recombinant strain for model calibration, which might have higher maintenance requirements, could further explain this result.

Finally, during controlled growth at the feed phase, neither ethanol nor arabitol are produced. All the carbon is directed towards biomass formation and the energy necessary for its synthesis and maintenance. This result agrees with previous fluxomic studies carried out in aerobic, glucose-limited chemostats [57, 58], where significant carbon fluxes through the oxidative and non-oxidative branches of the PPP were found, without arabitol formation. Furthermore, the model shows significant oxaloacetate transport from the cytosol to the mitochondria, which was also observed in the cited studies. The most distinguishable feature of this phase is the high activity of the TCA cycle, which almost doubles the flux through this pathway reported under glucose limited conditions in chemostats ([24, 57, 58]). This higher activity in the TCA is probably associated with the need to cope with maintenance and growth-associated energy requirements under stressful conditions, such as high cell density, especially when no significant substrate level phosphorylation besides glycolysis occurs.

This analysis could have been performed using the genome-scale model in static conditions by deriving instantaneous exchange rates from contiguous samples and determining the flux distributions by specific growth rate maximization. Nevertheless, the inspection of flux distributions after model calibration has the advantage of considering the overall behavior of the cells during the cultivations. This provides more experimental support for the determination of parameters such as ${m}_{ATP}$, ${K}_G$, that cannot be directly estimated but that have a strong impact on the model output.

#### Discovery of single knock-outs to improve recombinant Human Serum Albumin production using Minimization of Metabolic Adjustment (MOMA) as the objective function to simulate mutant behavior

We performed 670 (number of genes in the model) batch simulations of single knock-out strains to discover beneficial deletions for the production of recombinant Human Serum Albumin (HSA), a 66 kDa protein with 16 disulfide bridges, that comprises about one half of the total blood serum protein [81].

**Fig. 8:**
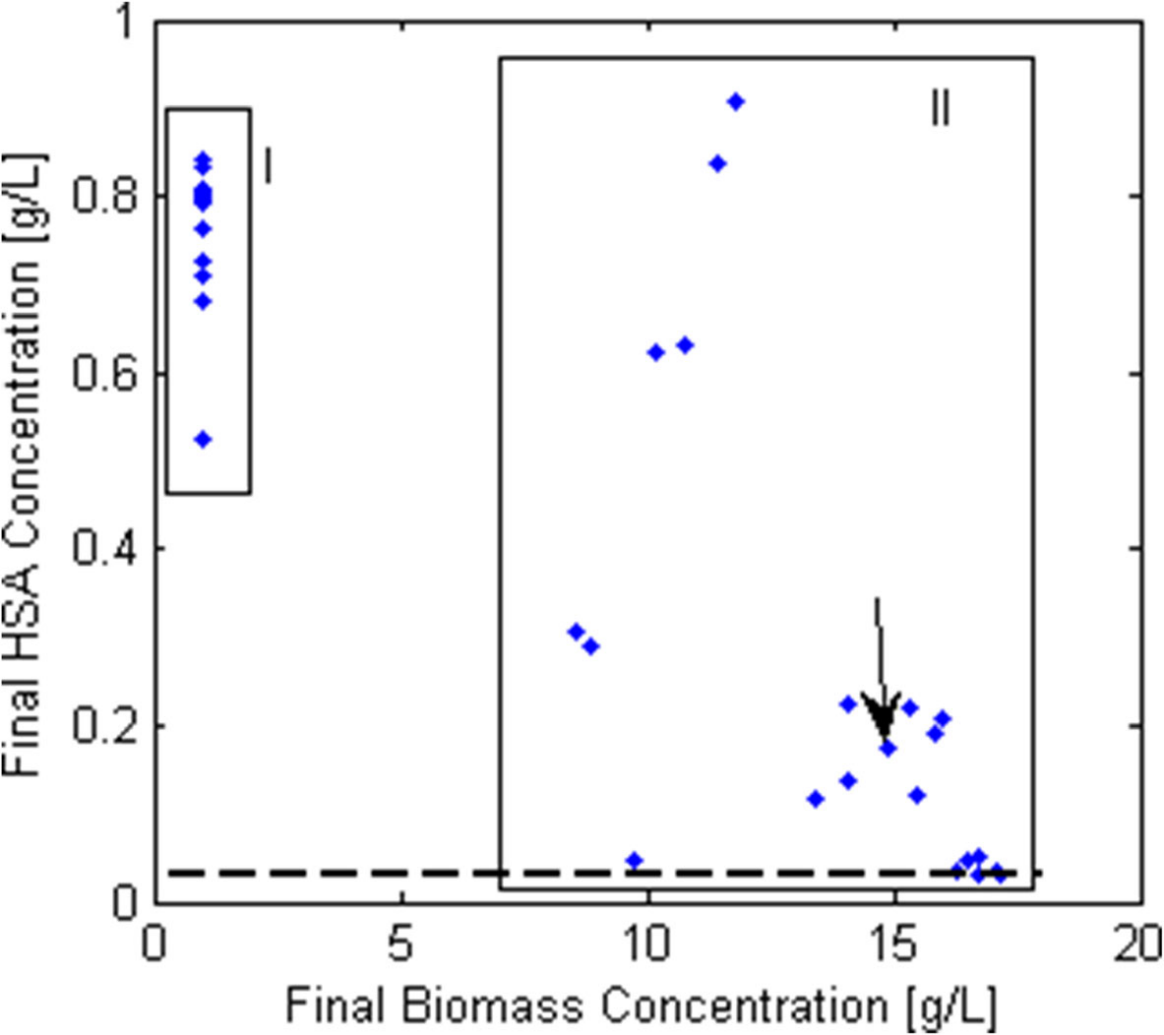
Final HSA vs. final biomass concentrations of simulated batch cultivations of single knock-out-strains. Blue dots correspond to the output of strains that improved the initial final HSA concentration (30 mg/L). Candidates out of Cluster II were manually analyzed. The red circle indicates the performance of the parental strain and the black arrow points to the methylene tetrahydrofolate dehydrogenase knock-out strain

The two main clusters (Fig. 8) show the relation between the final HSA and the final biomass concentration of the 130 mutations that improved HSA production (>30 mg/L at the end of the batch). The first cluster consists of strains that privilege HSA production over biomass formation; whereas the second one presents a trade-off between both.

We decided to leave Cluster I out of the analysis because of the impaired growth observed in the simulations, mainly due to the deletion of reactions associated to lipid biosynthesis. However, candidates from Cluster II (32 in total) were manually analyzed to identify the cause of HSA overproduction (Additional file 11).

**Fig. 9:**
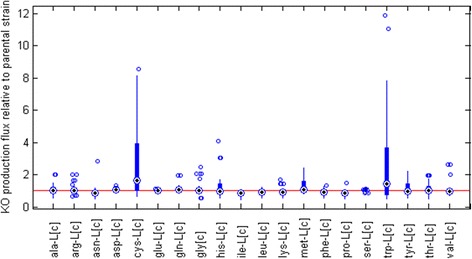
Turnover of key amino acids in knock-out strains relative to the parental strain. Each box summarizes how the production of each amino acid changed in the 32 knock out strains of Cluster II relative to the production in the parental strain (*Red Line*). Black dots correspond to the sample median, the extreme of the boxes to the 25^th^ and 75^th^ percentiles, the whiskers extend to the most extreme data points and circles mark outliers

A relative increase in the formation of cysteine and tryptophan was found for most of the candidates for Cluster II when compared to the parental strain, a trend that was not observed for the rest of the amino acids (Fig. 9). These energetically costly residues [82] are formed from serine. Therefore, re-routing carbon through this pathway could be beneficial to improve HSA production.

**Fig. 10:**
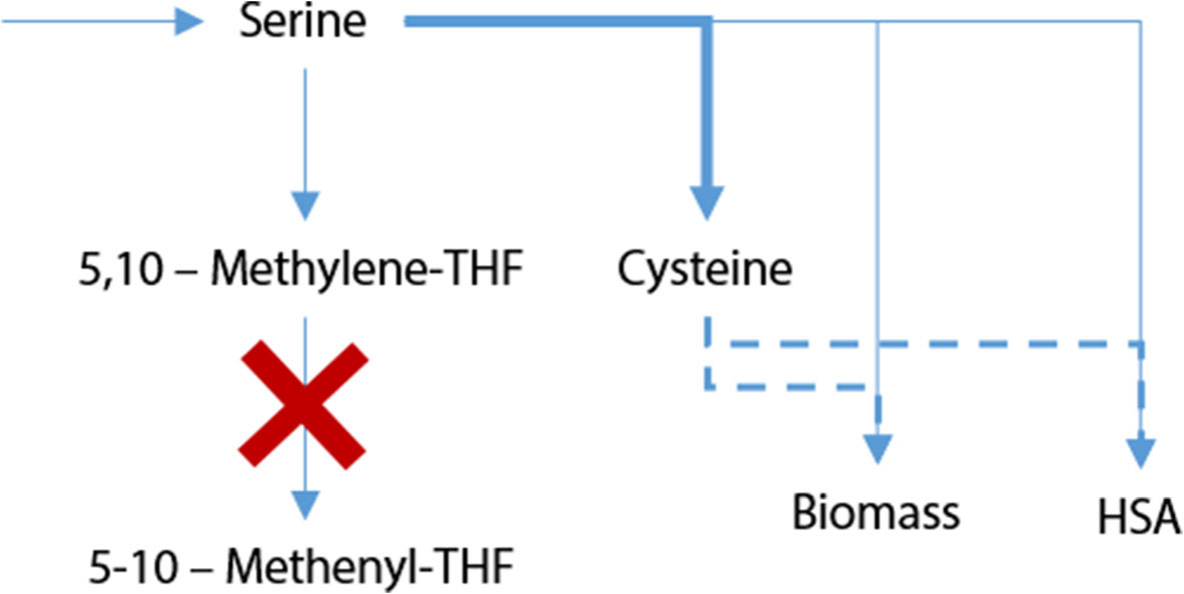
Rationale behind the knockout of the Methylene tetrahydrofolate (THF) dehydrogenase. By deleting this enzyme, the flux from Serine to 5-10-Methylene THF is blocked and redirected towards cysteine formation, whose availability increases the productivity of HSA

After manually analyzing the candidates, we found that one possible strategy could be the deletion of the cytosolic NAD-dependent methylene tetrahydrofolate dehydrogenase (Fig. 10). When compared to the parental strain, the knock-out results in a 6.3 fold improvement of the final concentration of the recombinant protein with a 5.8-fold increase in protein volumetric productivity (arrow in Fig. 8). This deletion eliminates the transformation of serine to 5–10 methylene tetrahydrofolate; hence, serine can be re-routed to two cysteine reactions. This gene is non-essential in *S. cerevisiae* [83] and, to the best of our knowledge, its essentiality has not been determined in *P. pastoris.* Therefore, it constitutes an interesting knock-out candidate to improve recombinant HSA production.

#### Bioprocess optimization for HSA overproduction

Here, we evaluated 13 feeding strategies of a fed-batch cultivation to improve the production of recombinant HSA. After the simulations, we selected a strategy that considered a slow decrease in the growth rate from μ = 0.14 h^−1^ to μ = 0.08 h^−1^ during the feeding phase (Table 9). The selected policy allows a 25% increase in volumetric productivity and reaches almost the same final HSA concentration as the constant growth rate strategy that reached the highest concentration (μ = 0.06 h^−1^).

**Table 9:** Feeding policies evaluated to improve the production of Serum Albumin in a particular bioreactor setup

Strategy	μ_MAX_	Rate	μ_MIN_	q_P_ [mg/g · h]	X_FINAL_ [g/L]	P_FINAL_ [mg/L]	Limitation
1	0.14	-	-	2.85	164.8	138	Oxygen
2	0.12	-	-	2.59	187.8	135	Oxygen
3	0.1	-	-	2.32	195.3	130	Volume
4	0.08	-	-	2.29	191.3	138	Volume
5	0.06	-	-	2.28	184.7	154	Volume
Best	0.14	0.1	0.08	2.83	197.5	150	Volume

This table shows the process indicators for the constant feeding Strategies (1–5) plus the best decreasing growth rate strategy. μ_MAX_ is the maximum growth rate in the feeding police. μ_MIN_ is the minimum growth rate in the feeding police. Rate is the rate of decreasing of set growth rate in feeding police. q_P_ is the protein productivity. X_FINAL_ and P_FINAL_ refer to the final concentration of biomass and serum albumin in the reactor when the simulation stops, which happened by either violating user-defined volume or Oxygen Transfer thresholds

The improvement in process productivity by modifying substrate addition during the feed phase is less efficient than the one attained by genetic modifications. However, other process variables such as reactor volume and oxygen transfer may be modified to further improve HSA production.

## Conclusions

Current GSMs of *P. pastoris* have been employed to address cellular behavior in stationary conditions. They have been successfully used for predicting production and consumption rates of different compounds and even achieving a 40% improvement of recombinant protein production by model-discovered knock-outs [42]. However, little attention has been given to the actual metabolic flux distribution that these reconstructions yield and how they evolve in a dynamic environment. Resulting flux distributions are important for two reasons: (i) they help to understand the cellular response to the different stresses to which the cell is subjected to and (ii) they can serve as input for several algorithms whose aim is to find metabolic engineering targets to improve the production of a certain compound.

In this work, we developed a robust dynamic GSM of glucose-limited aerobic cultivations of *P. pastoris*, linking and showing the impact that the model formulation process has over flux balance analysis. The assembled platform can fit several datasets with minimum significance, sensitivity and identifiability problems in its parameters. Moreover, if properly trained, it can be used to predict bioreactor dynamics. The model could also be employed to obtain realistic flux distributions throughout dynamic cultivations and to determine metabolic and process engineering strategies to improve the production of a target compound.

To broaden its applications to other relevant conditions for *P. pastoris*, the model could be calibrated with data from cultures with different carbon sources and feeding strategies, such as glycerol batch phase followed by a methanol induction phase. Also, the model could be used to study perturbations such as oxygen limitation, which is a common problem in industrial *P. pastoris* cultivations [84]. Moreover, it would be desirable to calibrate the model with data from a strain capable of producing high concentrations of a recombinant protein to understand and quantify the metabolic burden caused by this production.

Finally, it is expected that the incorporation of more curated metabolic reconstructions [44], gas mass balances and the knowledge derived from testing the hypotheses proposed using the model would improve its accuracy and broaden its applicability.

## Additional files

Additional file 1: Dynamic genome-scale metabolic model of *Pichia pastoris*. (RAR 11973 kb)
Additional file 2: Demonstration of the convexity of the solution space of the QP problem in the metabolic block. (DOCX 17 kb)
Additional file 3: Construction and evaluation of the iFS670 model. This file explains the modifications made on the iPP668 model to obtain the iFS670. Also, model performance is evaluated in terms of the internal flux distribution and the capacity of the model to predict experimental chemostat data in comparison to other available models at the beginning of the study. (DOCX 152 kb)
Additional file 4: iFS670 model for COBRA. (RAR 60 kb)
Additional file 5: Batch cultivation data used for model calibration, cross calibration, robustness check and validation. (XLSX 30 kb)
Additional file 6: Fed-Batch cultivation data used for model calibration, cross calibration, robustness check and validation. (XLSX 25 kb)
Additional file 7: Evaluation of Feeding policies. This file describes the performance of thirteen different feeding strategies on improving recombinant HSA production. (DOCX 23 kb)
Additional file 8: Cross Calibration Summary. Shows the performance of each one of the modeling structures derived using HIPPO (See Tables 3 and 5) after their calibration using the available datasets. (DOCX 107 kb)
Additional file 9: Absence of Parametric problems in the Robustness Check Datasets and Goodness of Fit. This file shows how the reduced (robust) modeling structures derived for the batch and fed-batch configurations presented no identifiability or sensitivity problems after being calibrated with new fermentation data. (DOCX 76 kb)
Additional file 10: Goodness of fit analysis of the validation datasets. (DOCX 95 kb)
Additional file 11: Knockout candidates for the overproduction of Human Serum Albumin. This file contains the details of the 32 candidates from Cluster II (Fig. 8) that could theoretically improve recombinant protein production. Expected final protein and biomass concentrations are reported. (DOCX 24 kb)
