# Supplementary material for: Dynamic genome-scale metabolic modeling of the yeast Pichia pastoris
Source: BMC Syst Biol. 2017 Feb 21;11:27. doi: 10.1186/s12918-017-0408-2 (PMC5320773; doi:10.1186/s12918-017-0408-2)
Supplement: Additional file 2: — Demonstration of the convexity of the solution space of the QP problem in the metabolic block. (DOCX 17 kb) [file 12918_2017_408_MOESM2_ESM.docx]

**Additional File 2: Demonstration of the convexity of the solution space of the QP problem in the metabolic block**

A convex optimization problem is one of the form:

$$Min f_{0}\left( x \right)$$

$$subject to f_{i}\left( x \right)\leq b_{i} i=1,\ldots,m$$

Where the functions $f_{0},\ldots,f_{m}:R^{n}\to R$ are convex, i.e., satisfy:

$f_{i}\left( \alpha x+\beta y \right)\leq\alpha f_{i}\left( x \right)+\beta f_{i}(y)$

For all x, y $\in R^{n}$ and all $\alpha, \beta\in R$ with $\alpha+\beta=1, \alpha\geq0, \beta\geq0$.

Now, for the problem solved in the metabolic block we have:

$$Min f_{0}\left( v \right): \alpha\cdot\sum v^{2}-(1-\alpha)\cdot\mu$$

$$subject to f\left( v \right): S\cdot v=b$$

Here, $v$ is an R^n^ flux distribution vector (also solution to the system), α is the suboptimal growth coefficient, μ is the specific growth rate of the cell (also a component of $v$), S is the stoichiometric matrix (m metabolites x n reactions) and b is the overall balance for each of the m metabolites of the network.

1. Demonstration of the convexity of $f_{0}\left( v \right): \alpha\cdot\sum v^{2}-(1-\alpha)\cdot\mu$

In order to determine the convexity of the problem, we have to demonstrate that:

$$f_{0}\left( \beta\cdot v_{1}+\gamma\cdot v_{2} \right)\leq\beta\cdot f_{0}\left( v_{1} \right)+\gamma\cdot f_{0}(v_{2})$$

$$\underset{I}{\underbrace{\alpha\cdot\sum\left( \beta\cdot v_{1}+\gamma\cdot v_{2} \right)^{2}-\left( 1-\alpha\right)\cdot\left( \beta\cdot\mu_{v_{1}}+\gamma\cdot\mu_{v_{2}} \right)}}\leq\underset{II}{\underbrace{\beta\cdot f_{0}\left( v_{1} \right)+\gamma\cdot f_{0}(v_{2})}}$$

Here, $v_{1},v_{2} \in R^{n}$ are any flux distribution vectors, $\beta+\gamma=1$, $\beta\geq0$, $\gamma\geq1$ . Now Expanding the expression above:

$$\alpha\cdot\sum\left( \beta^{2}\cdot v_{1}^{2}+2\cdot\beta\cdot\gamma\cdot v_{1}\cdot v_{2}+\gamma^{2}\cdot v_{2}^{2} \right)-\left( 1-\alpha\right)\cdot\left( \beta\cdot\mu_{v_{1}}+\gamma\cdot\mu_{v_{2}} \right)\leq\beta\cdot\underset{f_{0}(v_{1})}{\underbrace{\left( \alpha\cdot\sum v_{1}^{2}-\left( 1-\alpha\right)\cdot\mu_{v_{1}} \right)}}+\gamma\cdot\underset{f_{0}(v_{2})}{\underbrace{\left( \alpha\cdot\sum v_{2}^{2}-\left( 1-\alpha\right)\cdot\mu_{v_{2}} \right)}}$$

Which can be rearranged to:

$$\alpha\cdot\sum\left( \beta^{2}\cdot v_{1}^{2}+2\cdot\beta\cdot\gamma\cdot v_{1}\cdot v_{2}+\gamma^{2}\cdot v_{2}^{2} \right)-\left( 1-\alpha\right)\cdot\left( \beta\cdot\mu_{v_{1}}+\gamma\cdot\mu_{v_{2}} \right)\leq\alpha\cdot\left( \beta\cdot\sum v_{1}^{2}+\gamma\cdot\sum v_{2}^{2} \right)-(1-\alpha)\cdot\left( \beta\cdot\mu_{v_{1}}+\gamma\cdot\mu_{v_{2}} \right)$$

Eliminating equal terms

$$\alpha\cdot\sum\left( \beta^{2}\cdot v_{1}^{2}+2\cdot\beta\cdot\gamma\cdot v_{1}\cdot v_{2}+\gamma^{2}\cdot v_{2}^{2} \right)\leq\alpha\cdot\left( \beta\cdot\sum v_{1}^{2}+\gamma\cdot\sum v_{2}^{2} \right)$$

In our problem, $\alpha\geq0$. If α = 0, i.e. the metabolic block solves a LP problem, the inequality above is $0\leq0$, which is true, and therefore the problem is convex. If $\alpha>0$, it can be eliminated from the expression above:

$$\beta^{2}\cdot{\sum v}_{1}^{2}+2\cdot\beta\cdot\gamma\cdot\sum v_{1}\cdot v_{2}+\gamma^{2}\cdot\sum v_{2}^{2}\leq\beta\cdot\sum v_{1}^{2}+\gamma\cdot\sum v_{2}^{2}$$

Now, $\gamma=1-\beta$

$$\beta^{2}\cdot{\sum v}_{1}^{2}+2\cdot\beta\cdot\left( 1-\beta\right)\cdot\sum v_{1}\cdot v_{2}+\left( 1-\beta\right)^{2}\cdot\sum v_{2}^{2}\leq\beta\cdot\sum v_{1}^{2}+(1-\beta)\cdot\sum v_{2}^{2}$$

Rearranging the terms:

$$-\beta\cdot\left( 1-\beta\right)\cdot{\sum v}_{1}^{2}+2\cdot\beta\cdot\left( 1-\beta\right)\cdot\sum v_{1}\cdot v_{2}-\beta\cdot\left( 1-\beta\right)\cdot\sum v_{2}^{2}\leq0$$

Which can be reduced to:

$$\beta\cdot\left( 1-\beta\right)\cdot\sum\left( v_{1}-v_{2} \right)^{2}\geq0$$

According to the problem specifications, $\beta\geq0$ and $\beta+\gamma=1$, which constrains $\beta\in\left[ 0,1 \right]$. Also the term $\sum\left( v_{1}-v_{2} \right)^{2}$ is always positive or zero (if $v_{1}=v_{2}=\vec{0}$). Therefore, the inequality from above is always equal or larger than zero for the domain of the problem, confirming that the objective function is convex.

1. Demonstration of the convexity of $f\left( v \right): S\cdot v=b$

To demonstrate that the problem is convex, the restrictions for the optimization should be convex as well. Therefore, imitating the previous procedure:

$$f\left( \beta\cdot v_{1}+\gamma\cdot v_{2} \right)\leq\beta\cdot f\left( v_{1} \right)+\gamma\cdot f(v_{2})$$

$$S\cdot\left( \beta\cdot v_{1}+\gamma\cdot v_{2} \right)\leq\beta\cdot S\cdot v_{1}+\gamma\cdot S\cdot v_{2}$$

$$\beta\cdot S\cdot v_{1}+\gamma\cdot S\cdot v_{2}\leq\beta\cdot S\cdot v_{1}+\gamma\cdot S\cdot v_{2}$$

$$0\leq0$$

The last statement is true; therefore, the restrictions are also convex.

Finally, since the bi-objective function in the metabolic block and its restrictions are convex, the solution space of the problem is also convex. This ensures the optimality of the obtained flux distribution.
