# Supplementary material for: Dynamic genome-scale metabolic modeling of the yeast Pichia pastoris
Source: BMC Syst Biol. 2017 Feb 21;11:27. doi: 10.1186/s12918-017-0408-2 (PMC5320773; doi:10.1186/s12918-017-0408-2)
Supplement: Additional file 3: — Construction and evaluation of the iFS670 model. This file explains the modifications made on the iPP668 model to obtain the iFS670. Also, model performance is evaluated in terms of the internal flux distribution and the capacity of the model to predict experimental chemostat data in comparison to other available models at the beginning of the study. (DOCX 152 kb) [file 12918_2017_408_MOESM3_ESM.docx]

**Additional File 3 – Construction and evaluation of the iFS670 model**

The iFS670 model modified the iPP668 model from Chung *et al* [1]. in three aspects:

1. Contains stoichiometric equations for the production of recombinant Thaumatin, Human Serum Albumin (HSA) and FAB fragment.
2. Includes an NAD-dependent arabitol biosynthesis pathway
3. Reversibility from mitochondrial symporters and cytosolic reactions involving NAD/NADP was updated according to the suggestions from Pereira *et al* [2].

At the end of this Supplementary material we compare the performance of the iFS670 with two other models from *Pichia pastoris*.

1. **Stoichiometric reactions for the production of three recombinant proteins**

Thaumatin, HSA and FAB synthesis pathways were also included in the model according to the DNA, RNA and amino acid requirements employed in the iLC915 model [3] to form the primary structure of the protein:

| $0.997\cdot AA_{Prot}+0.0029\cdot RNA_{Prot}+0.000028\cdot DNA_{Prot}\to Protein [c]$ | ( 1 ) |
| --- | --- |
| $\left( \sum_{1}^{20} \beta_{i}\cdot aa_{i} \right)+\gamma\cdot ATP\left[ c \right]+\gamma\cdot H_{2}O\left[ c \right]\to AA_{Prot}$ | ( 2 ) |
| $\left( \sum_{1}^{4} \alpha_{i}\cdot NMP_{i} \right)+\delta\cdot ATP\left[ c \right]+\delta\cdot H_{2}O\left[ c \right]\to RNA_{Prot}$ | ( 3 ) |
| $\left( \sum_{1}^{4} \varepsilon_{i}\cdot dNMP_{i} \right)+\theta\cdot ATP\left[ c \right]+\theta\cdot H_{2}O\left[ c \right]\to RNA_{Prot}$ | ( 4 ) |

Coefficients for the different components of the proteins are detailed in Tables 1, 2 and 3. Codon usage was taken from [4] and was used as input for the calculation of RNA and DNA sequences online (<http://www.bioinformatics.org/sms2/rev_trans.html>).

**Table 1 - Amino acid requirements to form 1 gram of thaumatin, HSA and FAB fragment in the iPP669 model.** The coefficients here reported were used in equation 2, a cost of 4,3 mole of ATP was assumed per mole of amino acid assembles in the protein. All coefficients have mmol/gram of protein units.

| **Substrate** | **Thaumatin** | **HSA** | **Fab Fragment** |
| --- | --- | --- | --- |
| L-Alanine | 0,721 | 0,909 | 0,559 |
| L-Arginine | 0,541 | 0,390 | 0,430 |
| L-Asparagine | 0,451 | 0,245 | 0,215 |
| L-Aspartate | 0,541 | 0,519 | 0,387 |
| L-Cysteine | 0,721 | 0,505 | 0,215 |
| L-Glutamate | 0,271 | 0,895 | 0,473 |
| L-Glutamine | 0,180 | 0,289 | 0,559 |
| Glycine | 1,082 | 0,188 | 0,602 |
| L-Histidine | 0,000 | 0,231 | 0,172 |
| L-Isoleucine | 0,361 | 0,130 | 0,215 |
| L-Leucine | 0,406 | 0,923 | 0,774 |
| L-Lysine | 0,496 | 0,866 | 0,387 |
| L-Methionine | 0,045 | 0,101 | 0,043 |
| L-Phenylalanine | 0,496 | 0,505 | 0,387 |
| L-Proline | 0,541 | 0,346 | 0,516 |
| L-Serine | 0,631 | 0,404 | 1,376 |
| L-Threonine | 0,902 | 0,418 | 0,731 |
| L-Tryptophan | 0,135 | 0,029 | 0,086 |
| L-Tyrosine | 0,361 | 0,274 | 0,387 |
| L-Valine | 0,451 | 0,620 | 0,645 |
| ATP (γ) | 40,1 | 37,8 | 39,4 |

**Table 2 - RNA requirements for the production of 1 gram of Thaumatin, HSA and Fab fragment codifying RNA.** A cost of 2,4 mol of ATP per gram of RNA was assumed.

| **Substrate** | **Thaumatin** | **HSA** | **Fab Fragment** |
| --- | --- | --- | --- |
| AMP | 0,73 | 0,86 | 0,74 |
| UMP | 1,14 | 1,09 | 1,18 |
| GMP | 0,73 | 0,72 | 0,58 |
| CMP | 0,48 | 0,40 | 0,57 |
| ATP (δ) | 7,38 | 7,38 | 7,38 |

**Table 3 - DNA requirement for the formation of 1 gram of codifying DNA for Thaumatin, HSA and Fab Fragment.** A cost of 3,4 mol ATP per gram of DNA produced was assumed.

| **Substrate** | **Thaumatin** | **HSA** | **Fab Fragment** |
| --- | --- | --- | --- |
| dAMP | 1,14 | 0,86 | 1,18 |
| dTMP | 0,73 | 1,09 | 0,74 |
| dGMP | 0,48 | 0,72 | 0,57 |
| dCMP | 0,73 | 0,40 | 0,58 |
| ATP(θ) | 10,45 | 10,45 | 10,45 |

1. **Arabitol Biosynthesis Pathway**

In total, we added four reactions associated to this pathway [5]. First, Ribulose-5P is converted into D-Ribose by a kinase with the formation of ATP. Then, D-ribose is converted into D-arabitol by a dehydrogenase with the formation of NAD^+^ from NADH. After D-arabitol is synthesized, it is transported to the extracellular medium and then “consumed” by an exchange reaction (Figure 1).

**Figure 1 – D-Arabitol synthesis pathway from D-Ribulose-5-phosphate in Pichia pastoris.**


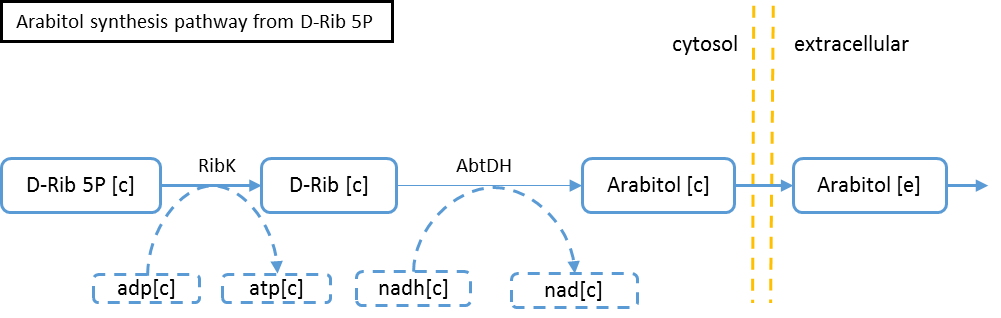


1. **Model manual curation**

Initially, the main problem of the model is that it did not carry flux through the oxidative part of the Pentose Phosphate Pathway (PPP), the main source of the reducing cofactor NADPH. Instead, this cofactor was synthesized by a cytosolic NADP-dependent isocitrate dehydrogenase, which was also the main source of α-ketoglutarate in the cytosol (data not shown). Fluxomic studies in aerobic glucose-limited conditions in *P. pastoris* [6]–[8], have shown that about 40% of the carbon that reaches glucose 6 phosphate is carried through the oxidative branch of the PPP, which is also thermodynamically favorable [9]. Moreover, α-ketoglutarate is considered to be produced in the mitochondria and then exported to the cytosol for nitrogen fixation and anabolic reactions.

These inconsistencies have been recently addressed for several genome scale metabolic models of *Saccharomyces cerevisiae* [2]. Therefore, we performed the following changes to our reconstruction according to the indication of the authors:

1. We enabled the transport of α-ketoglutarate from the mitochondria to the cytosol using transporters present in *P. pastoris* [10], [11].
2. The flux through three symporters that passively carried protons against the electrochemical gradient in the mitochondria was blocked in the direction of export to the cytosol.
3. Based on the assumption postulated by Satrustegui *et al* [12] for *Saccharomyces cerevisiae*, we considered that the NAD^+^/NADH and NADPH/NADP^+^ ratios in aerobic glucose-limited conditions are high enough to block the flux towards the formation of NAD^+^ and NADPH. Therefore, we blocked 30 cytosolic reactions in the direction of either NAD^+^ or NADPH formation. The only reactions left to produce cytosolic NADPH were the ones from the PPP and the acetate-forming acetaldehyde dehydrogenase, whose presence has been experimentally determined in aerobic glucose-limited cultivations of *P. pastoris* [13].

Applying these modifications resulted in a spontaneous flux through the PPP, a mitochondrial formation of α-ketoglutarate with its subsequent secretion to the cytosol and an overall concordance in the direction of the fluxes with respect to experimental data. This makes the model a reasonable approximation of *Pichia pastoris* central carbon metabolism.

When the flux distribution derived from the fed-batch robustness check dataset was compared with the fluxomic data obtained by Heyland *et al* [7] (equivalent conditions), the average error in the prediction of 23 fluxes of the central metabolism dropped three times with respect to the predictions made by the not curated model - from 128% to 39% for the exponential batch phase and from 160% to 63% for the controlled feed phase (Figure 2). This drop was mainly caused by the change in the direction (from negative to positive flux) of the non-oxidative part of the PPP, the spontaneous flux through the oxidative branch of this pathway and the reduction in the predicted influx of oxaloacetate to the cell. The overall agreement in directionality can be seen in Figure 2 by the elimination of negative predicted fluxes in the curated model.

**Figure 2 - Predicted versus experimental fluxes of the central metabolism.** The flux distributions determined by Heyland et al [7] during an aerobic glucose limited fermentation were compared to the output of the model in equivalent stages of a cultivation (exponential and controlled growth phases) during the experiment used for checking fed-batch model robustness. Values are presented as normalized to carbon uptake and the black line represents the unit.


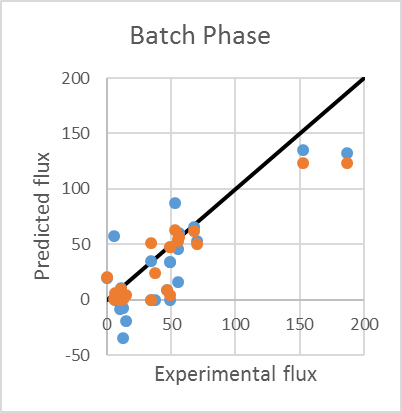

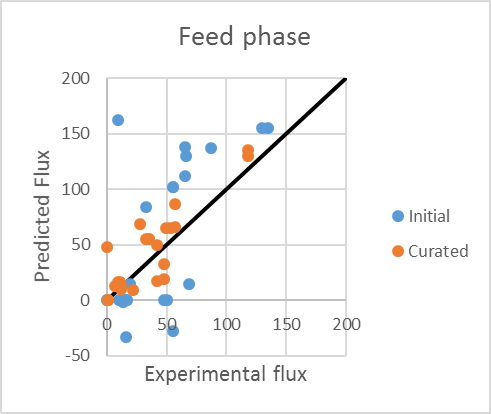


1. **Model Performance**

Usability and similarity to experimental chemostat data were used as criteria to select the most appropriate genome-scale model for building the dynamic framework. In terms of usability, we verified that the models had an adequate annotation, *i.e.* balanced equations, intuitive metabolite and reaction names, compartmentalization, functional gene-reaction associations and adequate representation of the central metabolism, among others. We then evaluated model similarity to experimental data from two chemostats (Table 1) using the normalized square differences between experimental and simulated rates (Equation 5):

| $F_{i}=\sum_{j=1}^{2} \frac{1}{n_{j}}\cdot\sum_{k=1}^{n_{j}} \frac{\sqrt{\left( v_{e{xp}_{k,j}}-v_{mod_{k,j}} \right)^{2}}}{v_{e{xp}_{k,j}}}$ | ( 5 ) |
| --- | --- |

Here, F is the overall fitting relative error of model i, n_J_ corresponds to the number of predicted rates determined in each dataset (12 in dataset 1 and 30 in dataset 2). Also, $v_{e{xp}_{k}}$ corresponds to the vector of experimental rates of condition k in dataset j and $v_{mod_{k,j}}$ is the model’s estimation of the experimental rates of condition k in dataset j.

For each prediction, we first constrained each model with n_J_-1 experimental rates. Then, Flux Balance Analysis (FBA) [14] was performed using biomass maximization as objective function to predict the remaining one.

It is worthy to note that whenever a model yielded an infeasible solution (due to carbon imbalance) or erroneously predicted the production of a compound under certain experimental condition, an error of 100% was assumed for that particular rate.

The model that gave best predictions compared to experimental data was chosen as the basis for the dynamic model. We tested the iFS670 model against three genome-scale metabolic models of *Pichia pastoris* that were available at the beginning of this study: the iPP668[1], the iLC915 [3] and the PpaMBEL1254 [15]

**Table 1 - Chemostat data used for model selection**

| **Set** | **Type of data** | **Rates** | **Conditions** | **Reference** |
| --- | --- | --- | --- | --- |
| 1 | Glycerol- and/or methanol-limited chemostats | 5 | 4 | [16] |
| 2 | Glucose-limited chemostats at different oxygen levels | 7 | 6 | [17] |

The main components and the relevant usability features of published GSMs of *Pichia pastoris* are detailed in Table 5. The PpaMBEL1254 model was discarded due to the lack of intuitive reaction and metabolite names in the online version, as well as the absence of gene-protein relations (at least in the online version), hampering the analysis of knock-out strains. All the models share the same structure of the central metabolism, which carries most of the flux entering the cell.

**Table 5 - Main components and usability features of available genome-scale metabolic models of Pichia pastoris**

|  | **iPP668** | **iFS670** | **PpaMBEL1254** | **iLC915** |
| --- | --- | --- | --- | --- |
| **Number of genes** | 669 | 670 | 540 | 915 |
| **Reactions** | 1354 | 1383 | 1254 | 1426 |
| **Metabolites** | 1177 | 1195 | 1058 | 1302 |
| **Compartments** | 8 | 8 | 8 | 6 |
| **Platform used for analysis** | Cobra | Cobra | Cobra | Raven |
| **Intuitive nomenclature for reactions and metabolites** | Yes | Yes | No | No |
| **Capable of performing Single Gene deletions** | Yes | Yes | No | Yes |
| **Capable of automatically checking mass balance** | No | No | No | Yes |

After the determination of the average relative error between model predictions and experimental data [16], [17] (Table 6), we selected the iFS670 model since it has a desirable structure and better reproduces experimental data from *P. pastoris* chemostats. It is worth mentioning that the inclusion of the arabitol biosynthesis pathway into Chung’s (iPP668) model – resulting in the iFS670 model - greatly improved the predictions of specific growth rate, Oxygen Uptake Rate (OUR) and Carbon Dioxide Evolution Rate (CER) in hypoxic glucose-limited chemostats (Figure 3 and Figure 4). Specifically, the deviation of carbon towards arabitol reduced the predicted growth rate in those conditions when compared to the iPP668 model, resulting in a reduction of the difference with the corresponding experimental value.

**Table 6 - Average error of model predictions using two datasets from carbon-limited chemostats.** In glycerol- and/or methanol (MetOH) – limited chemostats, the models were employed to predict specific growth rate µ, Oxygen Uptake Rate (OUR) and carbon dioxide evolution rate (CER) in four different conditions, which gives a total of 12 predictions. In the glucose limited chemostats, the models were used to estimate µ, OUR, CER, ethanol secretion rate and arabitol secretion rate in six conditions, which gives a total of 30 model predictions. Experimental data was taken from [16], [17]

| **Carbon Source** | **iLC915** | **iFS670** | **iPP669** | **Number of predictions** |
| --- | --- | --- | --- | --- |
| Glycerol/MetOH | 78% | 37% | 38% | 12 |
| Glucose | 85% | 36% | 52% | 30 |
| **Overall Error (F)** | 83% | 36% | 48% | 42 |

**Figure 3 - Experimental and model-predicted specific growth rates using glucose as the only carbon source at different oxygen levels for a P. pastoris wild type strain. Data taken from** [17]

**Figure 4 – Prediction of Gas exchange and secondary metabolite production by the tested models.** The percentage in the x axis correspond to oxygen fraction in the gas inlet of the bioreactor used to perform the study (21% 🡪 normoxic, 11% 🡪 oxygen limited, 8% 🡪 hypoxic). Data taken from

**Bibliography**

[1] B. K. S. Chung, S. Selvarasu, A. Camattari, J. Ryu, H. Lee, J. Ahn, H. Lee, and D. Lee, “Genome-scale metabolic reconstruction and in silico analysis of methylotrophic yeast Pichia pastoris for strain improvement,” *Microb. Cell Fact.*, vol. 9, no. 50, pp. 2–15, 2010.

[2] R. Pereira, J. Nielsen, and I. Rocha, “Improving the flux distributions simulated with genome-scale metabolic models of Saccharomyces cerevisiae,” *Metab. Eng. Commun.*, vol. 3, pp. 153–163, 2016.

[3] L. Caspeta, S. Shoaie, R. Agren, I. Nookaew, and J. Nielsen, “Genome-scale metabolic reconstructions of Pichia stipitis and Pichia pastoris and in silico evaluation of their potentials.,” *BMC Syst. Biol.*, vol. 6, no. 1, p. 24, Jan. 2012.

[4] K. De Schutter, Y.-C. Lin, P. Tiels, A. Van Hecke, S. Glinka, J. Weber-Lehmann, P. Rouzé, Y. Van de Peer, and N. Callewaert, “Genome sequence of the recombinant protein production host Pichia pastoris.,” *Nat. Biotechnol.*, vol. 27, no. 6, pp. 561–6, Jun. 2009.

[5] H. Cheng, J. Lv, H. Wang, B. Wang, Z. Li, and Z. Deng, “Genetically engineered Pichia pastoris yeast for conversion of glucose to xylitol by a single-fermentation process,” *Appl. Microbiol. Biotechnol.*, vol. 98, no. 8, pp. 3539–3552, 2014.

[6] M. Dragosits, J. Stadlmann, J. Albiol, K. Baumann, M. Maurer, B. Gasser, M. Sauer, F. Altmann, P. Ferrer, D. Mattanovich, and B. Cerdanyola, “The Effect of Temperature on the Proteome of Recombinant Pichia pastoris research articles,” *J. Proteome Res.*, no. 8, pp. 1380–1392, 2009.

[7] J. Heyland, J. Fu, L. M. Blank, and A. Schmid, “Quantitative physiology of Pichia pastoris during glucose-limited high-cell density fed-batch cultivation for recombinant protein production,” *Biotechnol. Bioeng.*, vol. 107, no. 2, pp. 357–368, 2010.

[8] K. Baumann, M. Carnicer, M. Dragosits, A. B. Graf, J. Stadlmann, P. Jouhten, H. Maaheimo, B. Gasser, J. Albiol, D. Mattanovich, and P. Ferrer, “A multi-level study of recombinant Pichia pastoris in different oxygen conditions.,” *BMC Syst. Biol.*, vol. 4, no. 1, p. 141, Jan. 2010.

[9] D. L. Nelson and M. M. Cox, *LEHNINGER, PRINCIPLES OF BIOCHEMISTRY*, Fifth Edit. New York: W. H. Freeman and company, 2008.

[10] M. Tomàs-Gamisans, P. Ferrer, and J. Albiol, “Integration and Validation of the Genome-Scale Metabolic Models of Pichia pastoris: A Comprehensive Update of Protein Glycosylation Pathways, Lipid and Energy Metabolism,” *PLoS One*, vol. 11, no. 1, p. e0148031, 2016.

[11] H. Rußmayer, M. Buchetics, C. Gruber, M. Valli, K. Grillitsch, G. Modarres, R. Guerrasio, K. Klavins, S. Neubauer, H. Drexler, M. Steiger, C. Troyer, A. Al Chalabi, G. Krebiehl, D. Sonntag, G. Zellnig, G. Daum, A. B. Graf, F. Altmann, G. Koellensperger, S. Hann, M. Sauer, D. Mattanovich, and B. Gasser, “Systems-level organization of yeast methylotrophic lifestyle,” *BMC Biol.*, vol. 13, no. 1, p. 80, 2015.

[12] J. Satrustegui, J. Bautista, and A. Machado, “NADPH/NADP+ Ratio - Regulatory Implications in Yeast Glyoxylic-Acid Cycle,” *Mol. Cell. Biochem.*, vol. 51, no. 2, pp. 123–127, 1983.

[13] J. Heyland, J. Fu, L. M. Blank, and A. Schmid, “Carbon metabolism limits recombinant protein production in Pichia pastoris.,” *Biotechnol. Bioeng.*, vol. 108, no. 8, pp. 1942–53, Aug. 2011.

[14] J. D. Orth, I. Thiele, and B. Ø. Palsson, “What is flux balance analysis?,” *Nat. Biotechnol.*, vol. 28, no. 3, pp. 245–8, Mar. 2010.

[15] S. B. Sohn, A. B. Graf, T. Y. Kim, B. Gasser, M. Maurer, P. Ferrer, D. Mattanovich, and S. Y. Lee, “Genome-scale metabolic model of methylotrophic yeast Pichia pastoris and its use for in silico analysis of heterologous protein production.,” *Biotechnol. J.*, vol. 5, no. 7, pp. 705–15, Jul. 2010.

[16] A. Solà, P. Jouhten, H. Maaheimo, F. Sánchez-Ferrando, T. Szyperski, and P. Ferrer, “Metabolic flux profiling of Pichia pastoris grown on glycerol/methanol mixtures in chemostat cultures at low and high dilution rates.,” *Microbiology*, vol. 153, no. Pt 1, pp. 281–90, Jan. 2007.

[17] M. Carnicer, K. Baumann, I. Töplitz, F. Sánchez-, D. Mattanovich, P. Ferrer, and J. Albiol, “Macromolecular and elemental composition analysis and extracellular metabolite balances of Pichia pastoris growing at different oxygen levels,” vol. 14, pp. 1–14, 2009.
