# Supplementary material for: Dynamic genome-scale metabolic modeling of the yeast Pichia pastoris
Source: BMC Syst Biol. 2017 Feb 21;11:27. doi: 10.1186/s12918-017-0408-2 (PMC5320773; doi:10.1186/s12918-017-0408-2)
Supplement: Additional file 7: — Evaluation of Feeding policies. This file describes the performance of thirteen different feeding strategies on improving recombinant HSA production. (DOCX 23 kb) [file 12918_2017_408_MOESM7_ESM.docx]

**Additional File 7 – Evaluation of feeding policies**

We tested 13 different feeding strategies, which yielded a constant or decreasing growth rate (Figure 1). The details of the strategies are presented in Table 21.

**Figure 1 – Constant (left) versus decreasing (right) growth rates during fed-batch culture**. Here, t_FEED_ corresponds to the time when the feed of the culture starts after batch cultivation. μ_MAX_, Rate and μ_MIN_ refer to the parameters used to describe the decreasing growth rate profile of each culture. We evaluated two values for each one of these parameters, which yielded eight dynamic feeding strategies (6-13 in Table 1).


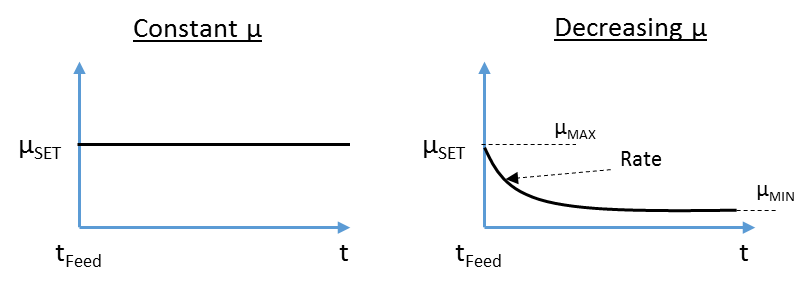


**Table 1 - Feeding strategies evaluated and productivity indicators.** The first five strategies attempt to make the culture grow at a constant growth rate while the rest produce a decreasing growth rate profile.

| **Strategy** | **μ_SET,MAX_ [h^-1^]** | **Rate** | **μ_SET,MIN_ [h^-1^]** | **q_P_ [mg/g_DCW_h]** | **X_FINAL_ [g/L]** | **P_FINAL_ [mg/L]** | **Limitation** |
| --- | --- | --- | --- | --- | --- | --- | --- |
| **1** | 0,14 | - | - | 2,85 | 164,8 | 138 | Oxygen |
| **2** | 0,12 | - | - | 2,59 | 187,8 | 135 | Oxygen |
| **3** | 0,1 | - | - | 2,32 | 195,3 | 130 | Volume |
| **4** | 0,08 | - | - | 2,29 | 191,3 | 138 | Volume |
| **5** | 0,06 | - | - | 2,28 | 184,7 | 154 | Volume |
| **6** | 0,14 | 0,07 | 0,08 | 2,13 | 193,1 | 121,6 | Volume |
| **7** | 0,14 | 0,07 | 0,04 | 1,33 | 176,6 | 92,3 | Volume |
| **8** | 0,14 | 0,01 | 0,08 | 2,83 | 197,5 | 150,0 | Volume |
| **9** | 0,14 | 0,01 | 0,04 | 2,34 | 195,1 | 128,0 | Volume |
| **10** | 0,1 | 0,07 | 0,08 | 1,88 | 191,0 | 111,6 | Volume |
| **11** | 0,1 | 0,07 | 0,04 | 0,89 | 172,8 | 66,9 | Volume |
| **12** | 0,1 | 0,01 | 0,08 | 1,41 | 193,7 | 81,3 | Volume |
| **13** | 0,1 | 0,01 | 0,04 | 2,30 | 188,8 | 140,5 | Volume |
