# Supplementary material for: Dynamic genome-scale metabolic modeling of the yeast Pichia pastoris
Source: BMC Syst Biol. 2017 Feb 21;11:27. doi: 10.1186/s12918-017-0408-2 (PMC5320773; doi:10.1186/s12918-017-0408-2)
Supplement: Additional file 8: — Cross Calibration Summary. Shows the performance of each one of the modeling structures derived using HIPPO (See Tables 3 and 5) after their calibration using the available datasets. (DOCX 107 kb) [file 12918_2017_408_MOESM8_ESM.docx]

**Additional File 8 – Initial Calibrations and parametric limitations**

Batch model

The model was used to calibrate data from eight aerobic, glucose-limited batch cultivations. The parameter values achieved in the calibrations is presented in Table 1, while the time of calibration and objective function value of the calibration are presented in Table 2. Datasets correspond to the following strains:

- Datasets 1 and 2: parental GS115 strain
- Datasets 3 and 4: recombinant strain harboring one copy of the thaumatin gene
- Datasets 5 and 6: recombinant strain harboring five copies of the thaumatin gene
- Datasets 7 and 8: recombinant strain harboring eight copies of the thaumatin gene

**Table 1 - Parameter values achieved in the calibration of data from eight batch cultivations using the initial batch model structure.**

|  | **Dataset** | | | | | | | | |
| --- | --- | --- | --- | --- | --- | --- | --- | --- | --- |
|  | **1** | **2** | **3** | **4** | **5** | **6** | **7** | **8** |  |
| $\boldsymbol{V}_{\boldsymbol{MAX}}$ | 7.75 | 3.34 | 7.95 | 2.74 | 3.07 | 6.67 | 1.51 | 1.27 |  |
| $\boldsymbol{K}_{\boldsymbol{S}}$ | 9.80E-04 | 9.60E-05 | 7e -4 | 3.00E-04 | 1.00E-05 | 1.5 e-4 | 1.00E-05 | 1.03E-05 |  |
| $\boldsymbol{v}_{\boldsymbol{EtOH,B}}$ | 1.98 | 1.78 | 1.76 | 1.48 | 0.89 | 2.97 | 0.03 | 0.02 |  |
| $\boldsymbol{v}_{\boldsymbol{Pyr,B}}$ | 0.21 | 0.18 | 0.25 | 0.19 | 0.12 | 0.2 | 0.01 | 0.003 |  |
| $\boldsymbol{v}_{\boldsymbol{Arab,B}}$ | 0.54 | 0.32 | 0.5 | 0.51 | 0.4 | 0.48 | 0.16 | 0.09 |  |
| $\boldsymbol{v}_{\boldsymbol{Cit,B}}$ | 0.06 | 0.04 | 0.08 | 1.1 | 0 | 0.05 | 0.03 | 0.05 |  |
| $\boldsymbol{\alpha}_{\boldsymbol{B}}$ | 4.05E-04 | 2.90E-04 | 4.20E-04 | 2.30E-04 | 1.45E-06 | 3.0E-04 | 7.19E-06 | 7.17E-05 |  |
| $\boldsymbol{m}_{\boldsymbol{atp}}$ | 0.52 | 0.68 | 0.001 | 4.09 | 9.99 | 4.61 | 3.29 | 1.32 |  |

**Table 2 - General features of initial batch model calibration.**

| **Dataset** | **N** | **Min. squares difference** | **Time of calibration [h]** |
| --- | --- | --- | --- |
| 1 | 8 | 0.26 | 1.92 |
| 2 | 8 | 1.55 | 5.29 |
| 3 | 8 | 0.47 | 3.46 |
| 4 | 8 | 0.88 | 4.40 |
| 5 | 6 | 1.69 | 4.11 |
| 6 | 9 | 7.27 | 4.41 |
| 7 | 12 | 1.17 | 4.32 |
| 8 | 13 | 3.30 | 4.30 |

We also provide two examples of how the model fitted two of these cultivations.

**Figure 1 - Batch model calibration of GS115 culture 1**

**Figure 2 - Batch model calibration of GS115 culture 8**


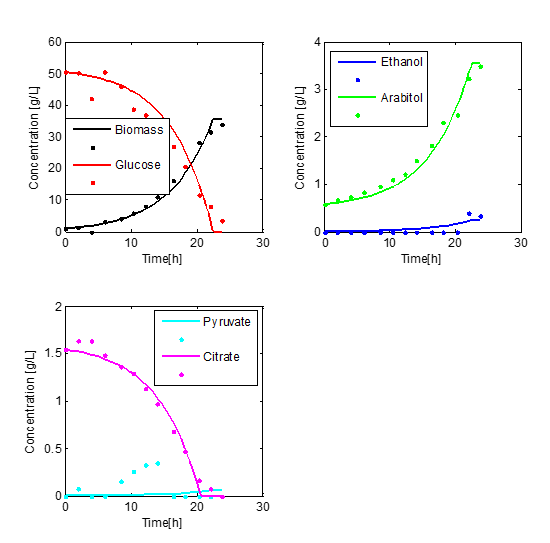


The recurrence of significance, sensitivity and identifiability issues found amongst the initial batch model calibrations is presented in Table 3 and Table 4.

**Table 3 – Percentage (o Frequency) of calibrations (8 in total) where a parameter presented sensitivity or significance issues. Parameters with recurrent problems are highlighted.**

|  | $V_{MAX}$ | $K_{S}$ | $v_{EtOH,B}$ | $v_{Pyr,B}$ | $v_{Arab,B}$ | $v_{Cit,B}$ | $\alpha_{B}$ | $m_{atp}$ |
| --- | --- | --- | --- | --- | --- | --- | --- | --- |
| **Sensitivity** | 0 | 38 | 0 | 0 | 0 | 13 | 25 | 13 |
| **Significance** | 25 | 50 | 0 | 13 | 0 | 25 | 25 | 25 |

**Table 4 - Percentage of calibrations (8 in total) where pairs of parameters show identifiability issues (correlation ≥ 0.95). Parameters with recurrent identifiability issues are highlighted.**

|  | $V_{MAX}$ | $K_{S}$ | $v_{EtOH,B}$ | $v_{Pyr,B}$ | $v_{Arab,B}$ | $v_{Cit,B}$ | $\alpha_{B}$ | $m_{atp}$ |
| --- | --- | --- | --- | --- | --- | --- | --- | --- |
| $V_{MAX}$ | - |  |  |  |  |  |  |  |
| $K_{S}$ | 13 | - |  |  |  |  |  |  |
| $v_{EtOH,B}$ | 50 | 0 | - |  |  |  |  |  |
| $v_{Pyr,B}$ | 25 | 0 | 38 | - |  |  |  |  |
| $v_{Arab,B}$ | 25 | 0 | 25 | 13 | - |  |  |  |
| $v_{Cit,B}$ | 13 | 0 | 0 | 13 | 0 | - |  |  |
| $\alpha_{B}$ | 25 | 25 | 0 | 0 | 0 | 13 | - |  |
| $m_{atp}$ | 63 | 25 | 38 | 25 | 38 | 13 | 50 | - |

Fed-batch model

Table 5 indicates the parameter values achieved for the three fed-batch cultivations used in the initial calibration of the fed-batch model. Figures 3, 4 and 5, show the model fits to the experimental data.

**Table 5 - Parameter Values of the Initial calibrations performed with the complete fed-batch model (14 parameters)**

| **Parameter** | **Dataset 1** | **Dataset 2** | **Dataset 3** | **Mean** | **Units** |
| --- | --- | --- | --- | --- | --- |
| $v_{S,max}$ | 2,74 | 3,29 | 2,59 | 2,94 | ${mmol}/{g_{DCW}h}$ |
| $K_{S}$ | 0,05 | 0,03 | 0,07 | 0,05 | $g/L$ |
| $v_{EtOH,B}$ | 1,95 | 2,18 | 0,98 | 1,58 | ${mmol}/{g_{DCW}h}$ |
| $v_{Pyr,B}$ | 0,18 | 0,18 | 0,13 | 0,15 | ${mmol}/{g_{DCW}h}$ |
| $v_{Arab,B}$ | 0,5 | 0,24 | 0,11 | 0,18 | ${mmol}/{g_{DCW}h}$ |
| $v_{Cit,B}$ | 0,12 | 0,11 | 0,22 | 0,16 | ${mmol}/{g_{DCW}h}$ |
| $v_{EtOH,FB}$ | 1,13 | 1,20 | 1,22 | 1,21 | ${mmol}/{g_{DCW}h}$ |
| $v_{Pyr,FB}$ | 0,10 | 0,02 | 0,26 | 0,14 | ${mmol}/{g_{DCW}h}$ |
| $v_{Arab,FB}$ | 0,07 | 0,13 | 0,17 | 0,15 | ${mmol}/{g_{DCW}h}$ |
| $v_{Cit,FB}$ | 0,005 | 0,00 | 0,01 | 0,008 | ${mmol}/{g_{DCW}h}$ |
| $\alpha_{B}$ | 3,03E-04 | 4,26E-05 | 1,49E-04 | 9,6E-05 | $\left[ - \right]$ |
| $\alpha_{FB}$ | 1,28E-04 | 2,22E-14 | 1,44E-04 | 7,2E-05 | $\left[ - \right]$ |
| $m_{ATP}$ | 4,38 | 9,00 | 8,13 | 8,6 | ${mmol}/{g_{DCW}h}$ |
| $T_{Fed}$ | 23 | 22,02 | 22,94 | 22,5 | $h$ |

**Table 6 - General features of initial batch model calibration.**

| **Dataset** | **N** | **Min. squares difference** | **Time of calibration [h]** |
| --- | --- | --- | --- |
| 1 | 22 | 3.84 | 29.05 |
| 2 | 21 | 4.35 | 30.75 |
| 3 | 22 | 4.17 | 29.21 |

**Figure 3 - Calibration of fed-batch dataset 1 using the original model structure**

**Figure 4 - Calibration of fed-batch dataset 2 using the original model structure**

**Figure 5 - Calibration of fed-batch dataset 3 using the original model structure**

The recurrence of significance, sensitivity and identifiability issues found amongst the initial fed-batch model calibrations is presented in Table 7 and Table 8.

**Table 7 - Percentage of times a parameter of the model presented sensitivity or significance problems out of a total of three model calibrations.** Parameters with sensitivity or significance issues are highlighted.

|  | $v_{S,max}$ | $K_{S}$ | $v_{Et,B}$ | $v_{Py,B}$ | $v_{Ar,B}$ | $v_{Ci,B}$ | $v_{Et,FB}$ | $v_{Py,FB}$ | $v_{Ar,FB}$ | $v_{Ci,FB}$ | $\alpha_{B}$ | $\alpha_{FB}$ | $m_{ATP}$ | $T_{Cons}$ |
| --- | --- | --- | --- | --- | --- | --- | --- | --- | --- | --- | --- | --- | --- | --- |
| **Sensitivity** | 0 | 0 | 0 | 0 | 0 | 0 | 0 | 0 | 0 | 33 | 0 | 67 | 0 | 0 |
| **Significance** | 0 | 33 | 0 | 0 | 0 | 0 | 0 | 33 | 0 | 100 | 67 | 100 | 0 | 0 |

**Table 8 – Frequency (in %) with which a pair of parameters presented identifiability issues in the initial modeling structure of fed-batch cultures of *Pichia pastoris* (3 datasets).** Parameters with recurrent identifiability issues are highlighted.

|  | $v_{S,max}$ | $K_{S}$ | $v_{EtOH,B}$ | $v_{Pyr,B}$ | $v_{Arab,B}$ | $v_{Cit,B}$ | $v_{EtOH,FB}$ | $v_{Pyr,FB}$ | $v_{Arab,FB}$ | $v_{Cit,FB}$ | $\alpha_{B}$ | $\alpha_{FB}$ | $m_{ATP}$ | $T_{Cons}$ |
| --- | --- | --- | --- | --- | --- | --- | --- | --- | --- | --- | --- | --- | --- | --- |
| $v_{S,max}$ |  |  |  |  |  |  |  |  |  |  |  |  |  |  |
| $K_{S}$ | 0 |  |  |  |  |  |  |  |  |  |  |  |  |  |
| $v_{EtOH,B}$ | 67 | 0 |  |  |  |  |  |  |  |  |  |  |  |  |
| $v_{Pyr,B}$ | 0 | 0 | 0 |  |  |  |  |  |  |  |  |  |  |  |
| $v_{Arab,B}$ | 33 | 0 | 33 | 0 |  |  |  |  |  |  |  |  |  |  |
| $v_{Cit,B}$ | 0 | 0 | 33 | 0 | 0 |  |  |  |  |  |  |  |  |  |
| $v_{EtOH,Fb}$ | 0 | 0 | 33 | 0 | 0 | 0 |  |  |  |  |  |  |  |  |
| $v_{Pyr,FB}$ | 0 | 0 | 0 | 0 | 0 | 0 | 0 |  |  |  |  |  |  |  |
| $v_{Arab,FB}$ | 0 | 0 | 33 | 0 | 0 | 33 | 33 | 0 |  |  |  |  |  |  |
| $v_{Cit,FB}$ | 0 | 0 | 0 | 0 | 0 | 0 | 0 | 0 | 33 |  |  |  |  |  |
| $\alpha_{B}$ | 0 | 0 | 0 | 0 | 0 | 0 | 0 | 0 | 33 | 0 |  |  |  |  |
| $\alpha_{FB}$ | 0 | 0 | 0 | 0 | 0 | 0 | 0 | 0 | 0 | 0 | 0 |  |  |  |
| $m_{ATP}$ | 0 | 0 | 0 | 0 | 0 | 0 | 0 | 0 | 0 | 0 | 0 | 0 |  |  |
| $T_{Cons}$ | 0 | 0 | 0 | 0 | 0 | 0 | 0 | 0 | 33 | 0 | 33 | 0 | 0 |  |
