# Supplementary material for: Dynamic genome-scale metabolic modeling of the yeast Pichia pastoris
Source: BMC Syst Biol. 2017 Feb 21;11:27. doi: 10.1186/s12918-017-0408-2 (PMC5320773; doi:10.1186/s12918-017-0408-2)
Supplement: Additional file 9: — Absence of Parametric problems in the Robustness Check Datasets and Goodness of Fit. This file shows how the reduced (robust) modeling structures derived for the batch and fed-batch configurations presented no identifiability or sensitivity problems after being calibrated with new fermentation data. (DOCX 76 kb) [file 12918_2017_408_MOESM9_ESM.docx]

**Additional File 9 – Absence of parametric problems in the Robustness Check Datasets and Goodness of Fit**

Batch Model

Table 1 and Table 2 show that the calibration of the Robustness Check dataset yielded no identifiability, sensitivity (correlation > 0.95 between parameters) and sensitivity issues. Figure 1 shows the results of the goodness of fit analysis for this dataset.

**Table 1 – Correlation Matrix of the robust parameter set used to calibrate the batch validation dataset.** Each cell contains the correlation between the two corresponding parameters.

|  | $\boldsymbol{v}_{\boldsymbol{EtOH,B}}$ | $\boldsymbol{v}_{\boldsymbol{Pyr,B}}$ | $\boldsymbol{v}_{\boldsymbol{Arab,B}}$ | $\boldsymbol{v}_{\boldsymbol{Cit,B}}$ | $\boldsymbol{\alpha}_{\boldsymbol{B}}$ |
| --- | --- | --- | --- | --- | --- |
| $\boldsymbol{v}_{\boldsymbol{EtOH,B}}$ | 1 | -0,45 | 0,43 | 0,78 | -0,86 |
| $\boldsymbol{v}_{\boldsymbol{Pyr,B}}$ | -0,45 | 1 | -0,17 | -0,56 | 0,50 |
| $\boldsymbol{v}_{\boldsymbol{Arab,B}}$ | 0,43 | -0,17 | 1 | 0,38 | -0,43 |
| $\boldsymbol{v}_{\boldsymbol{Cit,B}}$ | 0,78 | -0,56 | 0,38 | 1 | -0,88 |
| $\boldsymbol{\alpha}_{\boldsymbol{B}}$ | -0,86 | 0,50 | -0,43 | -0,88 | 1 |

**Table 2 - Sensitivity Matrix of the robust Parameter set used to calibrate the batch validation dataset. Each cell contains the average sensitivity of a particular parameter over the state variables.**

|  | **Volume** | **Biomass** | **Glucose** | **Ethanol** | **Pyruvate** | **Arabitol** | **Citrate** |
| --- | --- | --- | --- | --- | --- | --- | --- |
| $\boldsymbol{v}_{\boldsymbol{EtOH,B}}$ | 0 | 0,15 | 0,12 | 0,91 | 0,14 | 0,14 | 0,04 |
| $\boldsymbol{v}_{\boldsymbol{Pyr,B}}$ | 0 | 0,02 | 0,00 | 0,02 | 0,95 | 0,02 | 0,00 |
| $\boldsymbol{v}_{\boldsymbol{Arab,B}}$ | 0 | 0,03 | 0,01 | 0,02 | 0,02 | 0,97 | 0,00 |
| $\boldsymbol{v}_{\boldsymbol{Cit,B}}$ | 0 | 0,03 | 0,00 | 0,02 | 0,03 | 0,03 | 0,45 |
| $\boldsymbol{\alpha}_{\boldsymbol{B}}$ | 0 | 2,62 | 1,09 | 1,80 | 1,93 | 2,00 | 0,73 |


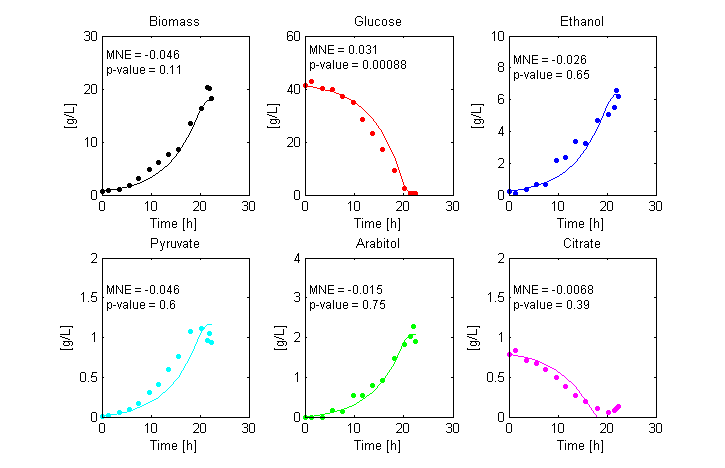


**Figure 1 - Goodness of fit of the batch model to the Robustness Check dataset**. This figure shows the model fit to experimental data along with the mean normalized error and the p-value of the Anderson-Darling test associated to each variable.

Fed-Batch Model

Table 3 and Table 4 indicate that the calibration using Structure 3 of new fed-batch data yielded no parametric problems. Figure 2 shows the results of the goodness of fit analysis for this dataset.

**Table 3 - Correlation Matrix of the calibration of the fed-batch validation dataset.** Recall that we left the parameters associated to Pyruvate dynamics out of the analysis since it was not produced during the cultivation

|  | $v_{MAX}$ | $K_{S}$ | $v_{Pyr,B}$ | $v_{Arab,B}$ | $v_{Cit,B}$ | $v_{Pyr,FB}$ | $\alpha_{B}$ | $\alpha_{FB}$ | $m_{ATP}$ | $T_{Cons}$ |
| --- | --- | --- | --- | --- | --- | --- | --- | --- | --- | --- |
| $v_{MAX}$ | 1 | 0,15 | - | -0,47 | 0,45 | - | -0,92 | 0,14 | -0,85 | 0,04 |
| $K_{S}$ | 0,15 | 1 | - | -0,85 | 0,62 | - | -0,20 | 0,68 | 0,24 | -0,32 |
| $v_{Pyr,B}$ | - | - | - | - | - | - | - | - | - | - |
| $v_{Arab,B}$ | -0,47 | -0,85 | - | 1 | -0,66 | - | 0,44 | -0,70 | 0,07 | 0,55 |
| $v_{Cit,B}$ | 0,45 | 0,62 | - | -0,66 | 1 | - | -0,51 | 0,60 | -0,20 | -0,10 |
| $v_{Pyr,FB}$ | - | - | - | - | - | - | - | - | - | - |
| $\alpha_{B}$ | -0,92 | -0,20 | - | 0,44 | -0,51 | - | 1 | -0,18 | 0,88 | -0,08 |
| $\alpha_{FB}$ | 0,14 | 0,68 | - | -0,70 | 0,60 | - | -0,18 | 1 | 0,21 | -0,42 |
| $m_{ATP}$ | -0,85 | 0,24 | - | 0,07 | -0,20 | - | 0,88 | 0,21 | 1 | -0,25 |
| $T_{Cons}$ | 0,04 | -0,32 | - | 0,55 | -0,10 | - | -0,08 | -0,42 | -0,25 | 1 |

**Table 4 - Sensitivity Matrix of the calibration of the fed-batch validation dataset.** All of the included parameters have a significant impact in at least one of the state variables.

|  | **Volume** | **Biomass** | **Glucose** | **Ethanol** | **Pyruvate** | **Arabitol** | **Citrate** |
| --- | --- | --- | --- | --- | --- | --- | --- |
| $v_{MAX}$ | 0,00 | 0,65 | 0,93 | 4,32 | 3,67 | 1,57 | 0,61 |
| $K_{S}$ | 0,00 | 0,22 | 0,00 | 0,01 | 1,24 | 0,02 | 0,00 |
| $v_{Pyr,B}$ | - | - | - | - | - | - | - |
| $v_{Arab,B}$ | 0,00 | 0,10 | 0,09 | 0,69 | 2,74 | 0,91 | 0,22 |
| $v_{Cit,B}$ | 0,00 | 0,03 | 0,00 | 0,09 | 0,96 | 0,04 | 0,62 |
| $v_{Pyr,FB}$ | - | - | - | - | - | - | - |
| $\alpha_{B}$ | 0,00 | 1,03 | 0,88 | 5,82 | 6,27 | 2,91 | 1,24 |
| $\alpha_{FB}$ | 0,00 | 0,03 | 0,00 | 0,01 | 0,00 | 0,02 | 0,00 |
| $m_{ATP}$ | 0,00 | 0,79 | 0,65 | 3,24 | 3,66 | 2,13 | 0,95 |
| $T_{Cons}$ | 0,00 | 0,26 | 0,00 | 0,22 | 3,66 | 3,06 | 0,88 |

**Figure 2 - Goodness of fit of the batch model to the Robustness Check dataset.** This figure shows the model fit to experimental data along with the mean normalized error and the p-value of the Anderson-Darling test associated to each variable.
