# Supplementary material for: Dynamic genome-scale metabolic modeling of the yeast Pichia pastoris
Source: BMC Syst Biol. 2017 Feb 21;11:27. doi: 10.1186/s12918-017-0408-2 (PMC5320773; doi:10.1186/s12918-017-0408-2)
Supplement: Additional file 10: — Goodness of fit analysis of the validation datasets. (DOCX 95 kb) [file 12918_2017_408_MOESM10_ESM.docx]

**Additional File 10: Goodness of Fit analysis of the Validation datasets**

Figures 1 and 2 present the mean normalized error and the p-value of the Anderson-Darling test for each state variable in the validation cultures for the batch and fed-batch models, respectively.

Batch model


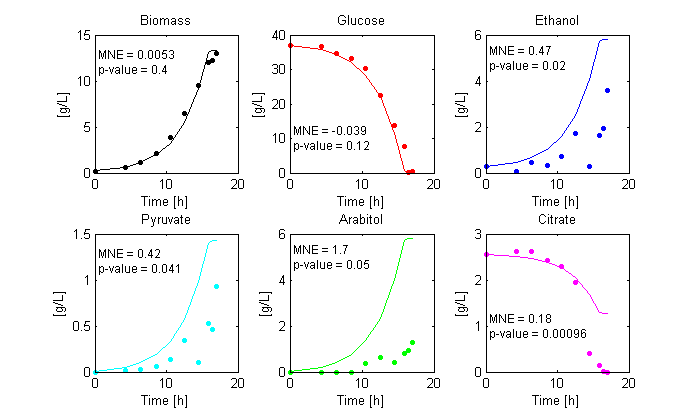


**Figure 1 - Goodness of fit analysis of the batch model prediction of the Validation dataset.**

Fed-batch model


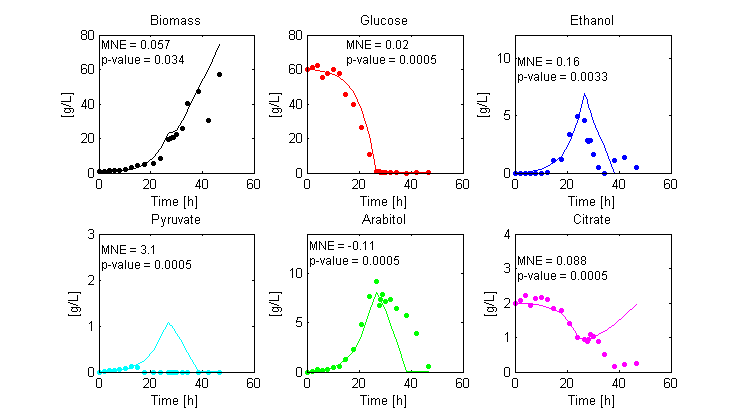


**Figure 2 - Goodness of fit analysis of the fed-batch model prediction of the Validation dataset.**
