# Supplementary material for: Dynamic genome-scale metabolic modeling of the yeast Pichia pastoris
Source: BMC Syst Biol. 2017 Feb 21;11:27. doi: 10.1186/s12918-017-0408-2 (PMC5320773; doi:10.1186/s12918-017-0408-2)
Supplement: Additional file 11: — Knockout candidates for the overproduction of Human Serum Albumin. This file contains the details of the 32 candidates from Cluster II (Fig. 8) that could theoretically improve recombinant protein production. Expected final protein and biomass concentrations are reported. (DOCX 24 kb) [file 12918_2017_408_MOESM11_ESM.docx]

**Supplementary Material 11 – Knockout Candidates derived using MOMA**

**Table 1 - Knockout candidates for HSA overproduction**

|  | **Deleted Gene** | **Final Biomass [g/L]** | **Final HSA [g/L]** | **Reaction(s) Name(s)** |
| --- | --- | --- | --- | --- |
| **1** | PAS_chr2-2_0094 | 11,81 | 0,91 | Chitin synthase |
| **2** | PAS_chr1-4_0194 | 11,41 | 0,84 | Putrescine and Spermidine transport |
| **3** | PAS_chr4_0836 | 11,41 | 0,84 | Putrescine and Spermidine transport |
| **4** | RPPA11109 | 10,74 | 0,63 | ribulose 5-phosphate 3-epimerase |
| **5** | RPPA11110 | 10,74 | 0,63 | ribulose 5-phosphate 3-epimerase |
| **6** | PAS_chr2-2_0330 | 10,20 | 0,62 | Phosphoryl ceramide syntase |
| **7** | PAS_chr2-2_0044 | 8,59 | 0,30 | CDP-Diacylglycerol synthetase, yeast-specific |
| **8** | PAS_chr4_0210 | 8,84 | 0,29 | ADP/ATP transporter, mitochondrial |
| **9** | PAS_chr4_0212 | 8,84 | 0,29 | ribose-5-phosphate isomerase |
| **10** | PAS_chr3_0604 | 14,05 | 0,23 | Deoxyribokinase and ribokinase |
| **11** | PAS_chr4_0408 | 15,33 | 0,22 | phosphoethanolamine cytidyltransferase |
| **12** | PAS_chr1-1_0418 | 16,02 | 0,21 | Acetate transporter |
| **13** | PAS_chr1-3_0220 | 15,88 | 0,19 | Methylenetetrahydrofolate dehydrogenase NAD |
| **14** | PAS_chr1-4_0487 | 14,85 | 0,17 | Succinate Dehydrogenase |
| **15** | PAS_chr2-2_0278 | 14,85 | 0,17 | Peptide alpha-N-acetyltransferase |
| **16** | PAS_chr3_1110 | 14,85 | 0,17 | Tyrosyl-tRNA synthetase, mitochondrial |
| **17** | PAS_chr4_0733 | 14,85 | 0,17 | Succinate Dehydrogenase |
| **18** | PAS_chr3_0646 | 14,04 | 0,14 | Phospholipase D, yeast-specific |
| **19** | PAS_chr3_0471 | 15,44 | 0,12 | aspartate-semialdehyde dehydrogenase, irreversible |
| **20** | PAS_chr2-1_0657 | 13,40 | 0,12 | phosphoglycerate dehydrogenase |
| **21** | PAS_chr4_0284 | 13,40 | 0,12 | ribonucleoside-diphosphate reductase |
| **22** | PAS_chr4_0877 | 16,73 | 0,05 | malate, succinate and fumarate transport, mitochondrial |
| **23** | PAS_chr3_0176 | 16,53 | 0,05 | N-acteylglutamate synthase and ornithine transacetylase , mitochondrial |
| **24** | PAS_chr1-1_0050 | 9,78 | 0,05 | Pyruvate dehydrogenase |
| **25** | PAS_chr1-4_0254 | 9,78 | 0,05 | Ppyruvate dehydrogenase |
| **26** | PAS_chr1-4_0593 | 9,78 | 0,05 | Pyruvate dehydrogenase, tetrahydrofolate aminomethyltransferase |
| **27** | PAS_chr2-2_0288 | 9,78 | 0,05 | Arginase |
| **28** | PAS_chr3_0649 | 16,26 | 0,03 | Thiamine transport in via proton symport |
| **29** | PAS_chr2-2_0127 | 17,10 | 0,03 | Cytochrome c peroxidase, mitochondrial |
| **30** | PAS_chr1-4_0659 | 17,10 | 0,03 | Hydrogen peroxide reductase thioredoxin, peroxisomal |
| **31** | PAS_chr2-1_0547 | 16,70 | 0,03 | 3',5'-bisphosphate nucleotidase |
| **32** | PAS_chr3_0462 | 17,14 | 0,03 | Alanyl-tRNA synthetase |

**Table 2 - Reactions and pathways associated to the deletion candidates**

|  | **Deleted Gene** | **Reactions** | **Pathway** |
| --- | --- | --- | --- |
| **1** | PAS_chr2-2_0094 | udpacgam[c] => h[c] + udp[c] + chitin[c] | Glutamate metabolism |
| **2** | PAS_chr1-4_0194 | h[c] + ptrc[e] => h[e] + ptrc[c]  h[c] + spmd[e] => h[e] + spmd[c] h[c] + sprm[e] => h[e] + sprm[c] | Transport, Extracellular |
| **3** | PAS_chr4_0836 | h[c] + ptrc[e] => h[e] + ptrc[c]  h[c] + spmd[e] => h[e] + spmd[c] h[c] + sprm[e] => h[e] + sprm[c] | Transport, Extracellular |
| **4** | RPPA11109 | ru5p-D[c] <=> xu5p-D[c] | Pentose Phosphate Pathway |
| **5** | RPPA11110 | ru5p-D[c] <=> xu5p-D[c] | Pentose Phosphate Pathway |
| **6** | PAS_chr2-2_0330 | ptd1ino_PP[c] + cer1_24[c] => 12dgr_PP[c] + ipc124_PP[c] | Sphingolipid Metabolism |
| **7** | PAS_chr2-2_0044 | h[c] + pa_PP[c] + ctp[c] <=> ppi[c] + cdpdag_PP[c]  h[m] + ctp[m] + pa_PP[m] <=> ppi[m] + cdpdag_PP[m] | Phospholipid Biosynthesis |
| **8** | PAS_chr4_0210 | h[c] + adp[c] + atp[m] => h[m] + atp[c] + adp[m] | Transport, Mitochondrial |
| **9** | PAS_chr4_0212 | r5p[c] <=> ru5p-D[c] | Pentose Phosphate Pathway |
| **10** | PAS_chr3_0604 | atp[c] + rib-D[c] => h[c] + adp[c] + r5p[c] | Pentose Phosphate Pathway |
| **11** | PAS_chr4_0408 | h[c] + ctp[c] + ethamp[c] => ppi[c] + cdpea[c] | Phospholipid Biosynthesis |
| **12** | PAS_chr1-1_0418 | ac[e] <=> ac[c] | Transport, Extracellular |
| **13** | PAS_chr1-3_0220 | nad[c] + mlthf[c] => nadh[c] + methf[c] | Folate Metabolism |
| **14** | PAS_chr1-4_0487 | fad[m] + succ[m] <=> fadh2[m] + fum[m]  q6[m] + succ[m] <=> q6h2[m] + fum[m]  q6[m] + fadh2[m] <=> q6h2[m] + fad[m] | Citric Acid Cycle/Oxydative Phosphorilation |
| **15** | PAS_chr2-2_0278 | accoa[c] + pepd[c] => h[c] + coa[c] + apep[c] | Other Amino Acid Metabolism |
| **16** | PAS_chr3_1110 | atp[m] + tyr-L[m] + trnatyr[m] => amp[m] + ppi[m] + tyrtrna[m] | tRNA charging |
| **17** | PAS_chr4_0733 | fad[m] + succ[m] <=> fadh2[m] + fum[m]  q6[m] + succ[m] <=> q6h2[m] + fum[m]  q6[m] + fadh2[m] <=> q6h2[m] + fad[m] | Citric Acid Cycle/Oxydative Phosphorilation |
| **18** | PAS_chr3_0646 | h2o[c] + pc_PP[c] => h[c] + pa_PP[c] + chol[c] | Phospholipid Metabolism |
| **19** | PAS_chr3_0471 | h[c] + nadph[c] + 4pasp[c] => pi[c] + nadp[c] + aspsa[c] | Alanine and Aspartate Metabolism |
| **20** | PAS_chr2-1_0657 | nad[c] + 3pg[c] => h[c] + nadh[c] + 3php[c] | Glycine and Serine Metabolism |
| **21** | PAS_chr4_0284 | 19 Reactions | Nucleotide Salvage Pathway |
| **22** | PAS_chr4_0877 | pi[m] + mal-L[c] <=> pi[c] + mal-L[m] | Transport, Mitochondrial |
| **23** | PAS_chr3_0176 | accoa[m] + glu-L[m] => h[m] + coa[m] + acglu[m]  glu-L[m] + acorn[m] => acglu[m] + orn[m] | Arginine and Proline Metabolism |
| **24** | PAS_chr1-1_0050 | nad[m] + coa[m] + pyr[m] => nadh[m] + co2[m] + accoa[m] | Glycolysis/Gluconeogenesis |
| **25** | PAS_chr1-4_0254 | nad[m] + coa[m] + pyr[m] => nadh[m] + co2[m] + accoa[m] | Glycolysis/Gluconeogenesis |
| **26** | PAS_chr1-4_0593 | udpacgam[c] => h[c] + udp[c] + chitin[c] | Glycolysis/Gluconeogenesis |
| **27** | PAS_chr2-2_0288 | h[c] + ptrc[e] => h[e] + ptrc[c]  h[c] + spmd[e] => h[e] + spmd[c] h[c] + sprm[e] => h[e] + sprm[c] | Arginine and Proline Metabolism |
| **28** | PAS_chr3_0649 | h[c] + ptrc[e] => h[e] + ptrc[c]  h[c] + spmd[e] => h[e] + spmd[c] h[c] + sprm[e] => h[e] + sprm[c] | Transport, Extracellular |
| **29** | PAS_chr2-2_0127 | ru5p-D[c] <=> xu5p-D[c] | Oxidative Phosphorylation |
| **30** | PAS_chr1-4_0659 | ru5p-D[c] <=> xu5p-D[c] | Other |
| **31** | PAS_chr2-1_0547 | ptd1ino_PP[c] + cer1_24[c] => 12dgr_PP[c] + ipc124_PP[c] | Cysteine Metabolism |
| **32** | PAS_chr3_0462 | h[c] + pa_PP[c] + ctp[c] <=> ppi[c] + cdpdag_PP[c]  h[m] + ctp[m] + pa_PP[m] <=> ppi[m] + cdpdag_PP[m] | tRNA charging |
